# Dissecting cellular states of infiltrating microenvironment cells in melanoma by integrating single-cell and bulk transcriptome analysis

**DOI:** 10.1186/s12865-023-00587-8

**Published:** 2023-12-12

**Authors:** Aiai Shi, Min Yan, Bo Pang, Lin Pang, Yihan Wang, Yujia Lan, Xinxin Zhang, Jinyuan Xu, Yanyan Ping, Jing Hu

**Affiliations:** 1 School of Intelligent Medicine and Biotechnology, Guilin Medical University, Guilin, 541100 Guangxi China; 2 Department of Immunology, College of Basic Medicine, Chongqing Medical University, Chongqing, 400010 China; 3 College of Bioinformatics Science and Technology, Harbin Medical University, Harbin, 150081 Heilongjiang China

**Keywords:** Cell states, Microenvironment, Immune phenotype, Immunotherapy, Melanoma

## Abstract

**Background:**

Cellular states of different immune cells can affect the activity of the whole immune microenvironment.

**Methods:**

Here, leveraging reference profiles of microenvironment cell states that were constructed based on single-cell RNA-seq data of melanoma, we dissected the composition of microenvironment cell states across 463 skin cutaneous melanoma (SKCM) bulk samples through CIBERSORT-based deconvolution of gene expression profiles and revealed high heterogeneity of their distribution. Correspondence analysis on the estimated cellular fractions of melanoma bulk samples was performed to identify immune phenotypes. Based on the publicly available clinical survival and therapy data, we analyzed the relationship between immune phenotypes and clinical outcomes of melanoma.

**Results:**

By analysis of the relationships among those cell states, we further identified three distinct tumor microenvironment immune phenotypes: “immune hot/active”, “immune cold-suppressive” and “immune cold-exhausted”. They were characterized by markedly different patterns of cell states: most notably the CD8 T Cytotoxic state, CD8 T Mixed state, B non-regulatory state and cancer-associated fibroblasts (CAFs), depicting distinct types of antitumor immune response (or immune activity). These phenotypes had prognostic significance for progression-free survival and implications in response to immune therapy in an independent cohort of anti-PD1 treated melanoma patients.

**Conclusions:**

The proposed strategy of leveraging single-cell data to dissect the composition of microenvironment cell states in individual bulk tumors can also extend to other cancer types, and our results highlight the importance of microenvironment cell states for the understanding of tumor immunity.

**Supplementary Information:**

The online version contains supplementary material available at 10.1186/s12865-023-00587-8.

## Introduction

In the last decade, the goal of cancer immunotherapies has become to selectively recover tumor-induced immune deficiency in the tumor microenvironment (TME), switching from “immune enhancement” to “immune normalization” [1]. The immune checkpoint blockade therapy (targeting PD1 or CTLA4) has been successfully applied in treating melanoma, lung cancer and kidney cancer, which acts on the dysfunction or exhaustion state of T cells [2, 3]. However, limited clinical responses are observed as in melanoma, despite the high response rate, most patients are untreatable. Therefore, it is very urgent and important to further understand tumor immunity.

The complexity of tumors is reflected by complicated interactions between immune, stromal and malignant cells [4]. The tumor microenvironment (TME) mainly comprises these non-tumor cells and is important in tumorigenesis and development. T cells are the most abundant and well-studied type in the TME of solid tumors, which may exert multifunctional roles [5]. CD8 + T cells with cytotoxic state induce antitumor immunity and prevent tumor growth, and high densities of CD8 + cytotoxic T cells are associated with good prognosis in various cancers [6]. However, the TME can suppress activated T cell responses. Tumor, myeloid and stromal cells could mediate the exhaustion of T cells through exciting co-inhibitory molecules (e.g., PD1, TIM-3, and CTLA4) on the T cell surface [4, 5]. T cells with regulatory (Treg) phenotype can suppress antitumor immunity by secreting immunosuppressive cytokines [5, 7] and indicate poor prognosis in many tumor types with its presence of high densities in the TME [8]. Tumor-associated macrophages (TAMs) are another immune population in the TME, they can either repress or induce antitumor immunity and tumor growth [5]. Two principal functional states of pro-inflammatory M1 and tissue reparative M2 in TAMs could inhibit tumor progression and facilitate tumor growth and metastasis, respectively [2]. A high density of M1 macrophages has been associated with a favourable prognosis in some cancers. Conversely, the density of M2 macrophages generally correlates with a poor prognosis in tumors, including breast cancer and primary melanoma [8]. Furthermore, B cells with regulatory (Breg) phenotype could suppress antitumor immunity and facilitate tumor development by regulating different cell types including T cells and TAMs and associated with poor prognosis [7, 9]. While, many studies show that the presence of a large number of tumor-infiltrating B cells collaborates with T cells to promote antitumor immunity and indicates a good prognosis in cancers [7, 10]. Altogether, different cellular states of immune cells in the TME play distinct roles in affecting antitumor immunity and patient prognosis. If one could map the cellular states of infiltrating microenvironment cells in individual tumors at patient cohort level, it would greatly strength our knowledge on tumor immunity and the difference among patients.

Nowadays, several computational methods have been developed to permit the in-silico deconvolution of complex cellular mixtures [11–13]. CIBERSORT was one of such methods [11] and has been combined with single-cell RNA-seq (scRNA-seq) data to accurately deconvolve the cellular composition of solid tumors [14, 15]. However, studies on deconvolving components of cellular functional states in the TME are still lagging. In this study, we leveraged CIBERSORT-based deconvolution combined with scRNA-seq profiles from melanoma samples, to map functional states of infiltrating microenvironment cells in skin cutaneous melanoma (SKCM) bulk samples from The Cancer Genome Atlas (TCGA). Our analysis of the relationships among these cell states within the TME identified three different immune phenotypes that were correlated with progression-free survival and had implications in response to immune checkpoint therapy, which can be valuable in guiding the choice of immunotherapy in melanoma patients.

## Materials and methods

### Unsupervised subclustering of the major cell types

**Fig. 1:**
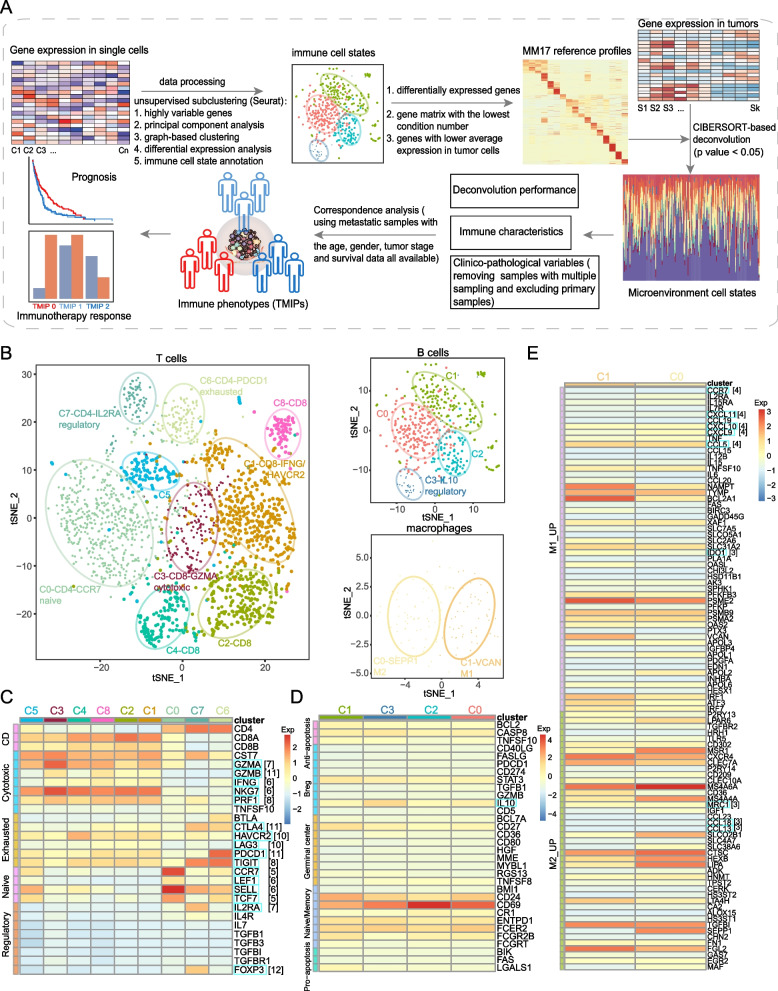
Dissection and clustering of tumor-related immune cells in melanoma. **A** Flow chart of the steps in the performed analyses. **B** The t-SNE projection of 2068 single T cells, 515 B cells and 126 macrophages from 19 patients, showing the formation of 9 main clusters of T cells, including 5 for CD8 + T cells and 3 for CD4 + T cells (left), 4 main clusters of B cells, including 1 for regulatory B cells and 3 for non-regulatory B cells (top right), and 2 main clusters of M1 and M2 macrophages (bottom right). Each dot corresponds to one single cell, colored according to cell cluster. **C** Heatmap showing the z-score normalized mean expression of selected T cell function-associated genes (row) in each T-cell cluster (C0-C8, column) of the 9 main clusters identified in (**B**). The T cell function-associated genes covering five different classes, including cytotoxic, exhausted, regulatory and naïve types. The T-cell clusters are colored according to those shown in (**B**). Blue boxes highlight the key markers and the numbers in brackets represent the total times appeared in literature. **D**-**E** Z-score normalized mean expression of the selected B cell function-associated genes (row) in each B-cell cluster (C0-C3, column) of the 4 main clusters (**D**) and z-score normalized mean expression of the selected macrophage function-associated genes (row) in each cell cluster (C0-C1, column) of the 2 main macrophage clusters (**E**). The B cell function-associated genes covering five different classes, including anti-apoptosis, regulatory B cell, germinal center, pro-apoptosis and naïve/memory types. The macrophage function-associated genes covering two different classes of M1 and M2 types. The B-cell and macrophage clusters are colored according to those shown in (**B**). Blue boxes highlight the key markers and the numbers in brackets represent the total times appeared in literature

The workflow of this study is shown in Fig. 1A. We obtained the single-cell RNA-Seq data of 4645 tumor-infiltrating cells from Tirosh and colleagues [4], GEO accession GSE72056. It corresponds to 19 melanoma patients and comprises primary tumors, lymph node metastasis or other lesions. Cell type identity was taken from Tirosh et al. (2016) (2068 T, 515 B, 52 NK cells, 126 macrophages, 61 CAFs, 65 endothelial cells, cancer cells as well as cells not assigned a specific cell type). Cells with < 1000 detected genes and genes without detected expression in any cells were filtered out. Totally, 4645 single cells and 20,079 genes remained and were included in downstream analyses. To identify subclusters within the three major non-malignant cell types (T, B and macrophage), we reanalyzed cells belonging to each of these three cell types separately using the Seurat R package (version 2.3.3) [16]. The FindVariableGenes function was used to determine the mean–variance relationship of the normalized counts of each gene across cells. We then chose genes whose log-mean and dispersion were above 1 as highly variable genes. The resulting variably expressed genes (1196 for T, 1233 for B, 1216 for macrophage) were summarized by principal component analysis, and the top principal components were selected by jackStraw procedure and PCElbowPlot function. Using the graph-based clustering approach implemented in the FindClusters function, with a conservative resolution of 0.8 and otherwise default parameters, each cell type was reclustered by its principal components. Totally 15 subclusters were identified. To visualize these clusters in two dimensions, these informative principal components were further summarized using tSNE dimensionality reduction of the RunTSNE function with its default settings.

After clustering results were obtained, we performed differential expression analysis using the Seurat FindAllMarkers function to define cell states for each of these 15 subclusters within these 3 cell types. Firstly, genes were claimed as differentially expressed if they have an average expression in that subcluster that was > twofold higher than the average expression in the other subclusters from that cell type, and a detectable expression in > 10% of all cells from that subcluster. Additionally, they were required to show statistical significance with *p* < 0.01 and bonferroni correction < 0.05. Finally, when naming the subclusters, we annotated the differentially expressed genes of each subcluster by known functional markers derived from the literatures (Table S1) and picked one representative gene indicative of the functional status of each subcluster from the top 100 ranked (based on fold change) differentially expressed genes. We also directly examined the expression levels of the known functional markers derived from the literatures (Table S1; [7, 9]). We surveyed the extant literatures and manually curated consensus lists of genes that were used in at least half of the literatures to be the key markers. The relevant literatures surveyed to determine these key marker genes were listed in Table S1 (below).

### Development of MM17 reference profiles

To dissect composition of cell states constituting melanoma tumor microenvironment (TME), we developed the MM17 reference profiles. Firstly, single-cell gene expression profiles (Tirosh et al. dataset) of all 18 cell subsets (15 immune cell subsets of T, B and macrophage, NK cells, CAFs and endothelial cells) of melanoma TME were utilized to derive signature genes for each subset. We tested the significance of differential expression for each gene detected in more than 10% of cells in each TME subset, comparing to all other subsets. Genes with log2(fold change) > 2 and FDR adjusted *p*-values < 0.01 based on Wilcoxon rank sum test were considered as significantly differentially expressed genes. Secondly, we filtered signature genes for each subset according to the condition number measure as previously described [11]. Briefly, significant genes of each TME subset were sorted by fold changes comparing to other cell subsets in a decreasing order. We iteratively selected the top 50 to 200 genes across all subsets, and constructed corresponding signature matrices by calculating average expression (non-log TPM values) within each cell subset. We retained the signature matrix with the lowest condition number which composed of 64 ~ 102 signature genes from each subset. Thirdly, to avoid potential confounding effects from tumor cells, we discarded those genes with higher average expression in tumor cells. During this process, the number of signature genes of C5 in T cells reduced dramatically from 102 to 9. This indicated significant confounding in this subset from tumor content, and 9 signature genes were not sufficient to represent a cell subset, thus we discarded this subset and its specific signature genes. Finally, 1351 genes were retained to construct the reference profile for deconvolution of 17 TME subsets (Table S2). Among these, the T cell clusters C1-CD8-IFNG/HAVCR2, C2-CD8, C4-CD8 and C8-CD8 expressing both cytotoxic and exhausted T cell markers were grouped as T_CD8_Mixed (Cytotoxic and Exhausted) state. The B cell clusters C0, C1 and C2 expressing non-regulatory B cell markers were grouped as the B_Non-regulatory (IL10-) state. The remaining clusters of T, B and macrophage were denoted by their corresponding states respectively as described in the text.

### Deconvolution of tumor microenvironment cell states

CIBERSORT [11] algorithm (with 100 permutations, disabling quantile normalization) was used to deconvolve compositions of TME cell states in bulk tumor samples using transcriptomic profiles (non-log TPM values). Here, we calculated cell fractions of T_CD8_Mixed state by aggregating those of T cell clusters C1-CD8-IFNG/HAVCR2, C2-CD8, C4-CD8 and C8-CD8, and B_Non-regulatory state by aggregating the three corresponding B cell clusters. Briefly, CIBERSORT utilized a novel application of linear support vector regression (SVR), which was highly robust with respect to noise, to deconvolve the bulk samples, and calculated an empirical p value for each sample to estimate the significance of presence of cell types in the reference profile. Only samples with *p* value < 0.05 were retained for analysis. For scRNA-seq datasets, we computed the artificial bulk expression data by calculating the average expression levels of each gene across all cells from the same sample.

### Alternative gene signatures

To explore the impact of signature gene selection on deconvolution performance, we compared the MM17 signature genes with three other signature gene sets and their combinations. The LM22 gene set was derived from Newman et al. [11], which denoted signature genes for 22 human normal leukocyte subsets. The gene sets (supp.3 and supp.12) were derived from supplementary tables by Tirosh et al. [4]. The supp.3 gene set consisted of genes differentially expressed among major cell types in melanoma microenvironment, including T cells, B cells, macrophages, natural killer cells, cancer-associated fibroblasts, endothelial cells and tumor cells. The supp.12 gene set contained genes preferentially expressed in regulatory T cells. Another three gene sets were derived by combining the above gene sets. The Merged gene set were the combination of LM22, supp.3 and supp.12. The LM22_MM17 gene set were the union of LM22 and MM17 gene sets. And The Merged_MM17 were the combination of Merged and MM17 gene set. Finally, these gene sets were used to construct corresponding reference profiles with the same cell states as MM17 reference profile, and then deconvolution results were compared with the actual fractions. Due to the extremely high proportions of unknown content in samples 59 and 78, which was not suitable for deconvolution [11], we discarded these two samples when estimating deconvolution performance.

### Validation of deconvolution performance using external scRNA-seq data

To further validate deconvolution performance of MM17 reference profiles, we applied it to an external scRNA-seq data of melanoma [17]. We obtained the processed expression profiles of TME cells directly from GEO database [18] under accession number GSE115978, and utilized a three-step approach. First, we trained an SVM classifier, using the caret R package (version 6.0–81) [19], based on expression levels of MM17 gene signatures and assign cell state labels for each cell in the Tirosh et al. data, which showed high accuracy of 0.87 (Figure S3D). Then, we applied this classifier to the new scRNA-seq data to assign each TME cell a cell state, and calculated the actual fractions of cell states in the new data set. Finally, deconvolution results of the new data set based on MM17 reference profiles were compared with the actual fractions.

### TCGA data

Transcriptomic profiles (TPM values) of 472 skin cutaneous melanoma (SKCM) samples from TCGA were downloaded (https://osf.io/gqrz9/wiki/home/; [20]). The clinical data of these samples was obtained from the Pancancer Atlas publication page (https://gdc.cancer.gov/about-data/publications/pancanatlas, Table S3), including the survival data, age at diagnosis, gender, race, American Joint Committee on Cancer (AJCC) pathologic tumor stage and tumor status. We also obtained the published molecular subtypes and immune characteristics of melanoma samples ([21]; https://gdc.cancer.gov/about-data/publications/panimmune).

### Identification of immune phenotypes and prognostic analysis

We performed correspondence analysis (CA) on a frequency table, in which each row represents a patient and each column represents the frequency of a cell state in that patient. Calculation of the projections, variance explained, and absolute contributions was performed by using the R packages FactoMineR (version 1.42) [22] and factoextra (version 1.0.5) [23]. The first two CA components of CA-1 and CA-2, accounting collectively for 47% of the variance in the co-association structure of the data, were used to identify three tumor microenvironment immune phenotypes (TMIPs): TMIP 0, TMIP 1 and TMIP 2 based on their median scores in patients. Accordingly, SKCM patients were separated into three groups with their corresponding TMIPs. Median value difference of cell state fraction among TMIPs was evaluated using Mood’s test. Furthermore, the difference of cell state fraction between any two TMIPs was computed using the two-sided Wilcoxon rank sum test. Associations between TMIP and survival were tested using univariate/multivariate Cox proportional hazard model that included age, sex, tumor stage, and TMIP as independent variables in the survival model and then Kaplan–Meier survival curve analysis with a log-rank comparison.

### Analysis of immunotherapy response

Therapy and response information of SKCM samples in TCGA were obtained from GDC data portal (https://portal.gdc.cancer.gov/; [24]). We extracted patients with measure_of_response information and with therapy_type as immunotherapy to explore associations between architectures of microenvironment components and response to immunotherapy, and we further extended this analysis to all patients with measure_of_response information. Patients with complete or partial response or stable disease state were defined as responders, and patients with progressive disease were defined as non-responders.

An additional dataset containing pre-treatment and on-treatment transcriptomic profiles and response to anti-PD1 (nivolumab, Nivo) therapy in melanoma (Table S6) was derived from Riaz [25]. As introduced by the authors, all patients received Nivo (3 mg/kg every 2 weeks) until progression or for a maximum of 2 years. All patients underwent biopsy before initial therapy (1–7 days before first dose of therapy, pre-treatment) and a repeat biopsy on cycle 1, day 29 (between days 23–29, on-treatment). Among these patients, a part of patients received anti-PD1 as the first-line treatment (ipilimumab (Ipi)-naive) while the others received anti-PD1 treatment after progression on prior anti-CTLA4 treatment (Ipi-progressed). Tumor response to Nivo for patients was defined by Response Evaluation Criteria in Solid Tumors (RECIST) v1.1 criteria. Patients with complete response (CR) or partial response (PR) or stable disease (SD) state were defined as responders, and patients with progressive disease (PD) were defined as non-responders. We first performed deconvolution to obtain cell state proportions of each patient, based on which we predicted the CA-1 and CA-2 scores using the R package FactoMineR. Then these patients were classified into the three groups with different architectures using the same thresholds as the TCGA data. The associations between architectures of microenvironment components and response to immunotherapy were examined using Fisher’s exact test. Moreover, we also tested the associations of the architectures with survival outcome of the patients using log-rank test.

## Results

### Identifying functional states of immune cells in the melanoma microenvironment

Single-cell RNA-seq profiles of non-malignant cells have highlighted the composition of the TME [4, 7]. Several studies have identified diverse functional subsets with specific expression states of the immune cell through finer clustering [26, 27]. To explore the functional states of the immune cells in the TME, we used the single-cell RNA-Seq data from 19 melanoma samples [4]. It includes 4645 cells and the majority have been assigned to known cell lineages: T cells (*n* = 2068), B cells (*n* = 515), natural killer (NK) cells (*n* = 52), macrophages (*n* = 126), cancer-associated fibroblasts (CAFs) (*n* = 61), endothelial (Endo) cells (*n* = 65) and cancer cells.

To identify subclusters within each of the three major non-malignant cell types, powered by their relatively large numbers in the dataset, we performed unsupervised clustering of cells using the graph-based clustering method implemented in Seurat [16] within each cell type (see Materials and methods). A total of 15 stable subclusters were identified, including 9 clusters for T, 4 clusters for B cells and 2 clusters for macrophages, each with its unique signature genes (Fig. 1B and Fig. S1A-E), revealing the expression heterogeneity. Importantly, when comparing across patients, these cell subclusters mostly consisted of cells from ten or more patients, indicating the diverse expression states in the TME are reproducibly detected across melanoma tumors and may thus represent common features of the melanoma TME, while they do vary in their proportions (Fig. S1C-F).

Reclustering of the T cells revealed 9 clusters (Fig. 1B and C; Fig. S2A and B). Cells of the first T cluster, C0-CD4-CCR7 (T_CD4_Naive) specifically expressed “naïve” marker genes such as CCR7, LEF1 and SELL. C6-CD4-PDCD1 (T_CD4_Exhausted) specifically expressed PDCD1 and BTLA, suggestive of the identity of exhausted CD4 T cells. C7-CD4-IL2RA (T_CD4_Regulatory), expressed high levels of markers IL2RA, FOXP3 and CTLA4, thus representing regulatory T cells. The fourth cluster, C3-CD8-GZMA (T_CD8_Cytotoxic), was characterized by the high expression of the cytotoxic molecules, including GZMA, NKG7 and GZMB, indicative of the status of cytotoxic CD8 T cells. C1-CD8-IFNG/HAVCR2 (T_CD8_Mixed), shared a few common genes with cluster 3, such as NKG7 and GZMB, but also with the HAVCR2, LAG3 and PDCD1 expression signatures that were associated with exhausted state, indicating the presence of possible partially exhausted T cells [28]. Furthermore, C2-CD8, C4-CD8 and C8-CD8, characterized by the high expression of NKG7, PRF1, and GZMA, also respectively expressed HAVCR2, TIGIT and PDCD1, HAVCR2 and TIGIT, HAVCR2, PDCD1 and TIGIT, indicating the diverse phenotypes of possible partially exhausted T cells (Fig. 1B and C; Fig. S2A-C).

Similarly, the B cells and macrophages were partitioned into 4 and 2 subsets, respectively. Among them, one subset (C3-IL10, B_Regulatory) of B cells expressed the classical marker IL10, indicating a regulatory B cell phenotype (Fig. 1B and D; Fig. S2D and E). The other three B cell clusters (C0, C1 and C2) expressing non-regulatory B cell markers were denoted as the B_Non-regulatory (IL10-) state. One subset of macrophages, C0-SEPP1 (M_M2), expressed high levels of many M2-type genes such as SEPP1, SLC38A6 and MSR1. The other subset, C1-VCAN (M_M1) was characterized by the high expression of the M1-type genes such as VCAN, NAMPT and BCL2A1 (Fig. 1B and E; Fig. S2D, F and G). Taken together, these results demonstrate that immune cells in the melanoma microenvironment present diverse functional states.

### Landscape of microenvironment cell states in bulk melanoma

The composition of the tumor immune microenvironment and the functional states of immune cells are key aspects for understanding of anti-cancer immune mechanism. Recent studies have explored the composition of main immune cell subpopulations in cancers using CIBERSORT [14, 29–31]. Here, we attempted to investigate the cellular functional states of infiltrating microenvironment cells in melanoma bulk tumors. Having identified the diverse immune cell states by single-cell RNA-seq data, we first identified signatures characterizing those cell states and evaluated the ability of these signatures to accurately deconvolve mixtures of cell states. Then, we constructed the map of microenvironment cell states in melanoma bulk samples using CIBERSORT. Finally, the association between these cell states and clinico-pathological variables was tested.

### Evaluation of deconvolution performance for microenvironment cell states

**Fig. 2:**
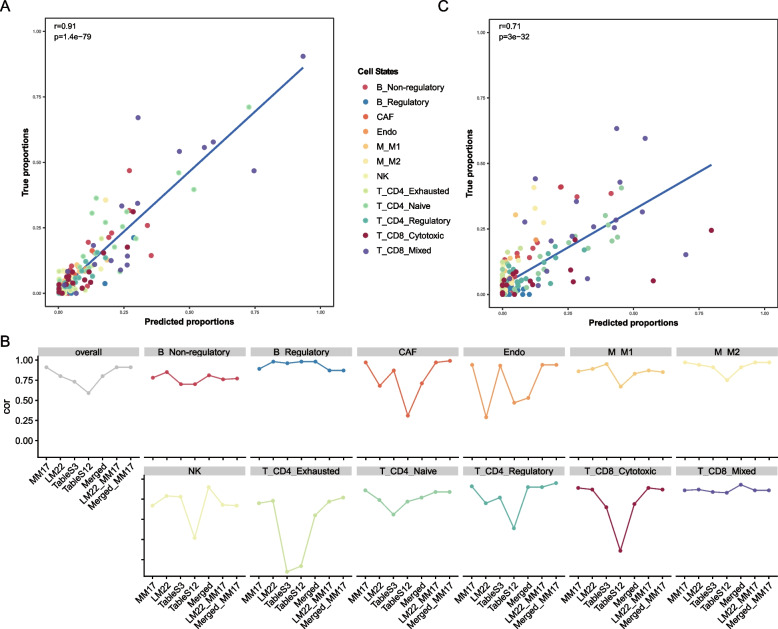
Performance assessment of MM17 on deconvolution of tumor microenvironment (TME) cell states. **A** Comparison between predicted proportions by deconvolution and true proportions by counting cells from scRNA-seq data (Tirosh et al*.).***B** Consistency between predicted proportions and true proportions when using different signature gene sets. **C** Consistency between predicted proportions and true proportions using an external scRNA-seq data from Jerby-Arnon et al*.* The dots in (**A**) and (**C**) represent corresponding cell proportions in patient tumors

To facilitate analyzing the cellular states of microenvironment cells in bulk melanoma tumors, we designed reference profiles (termed MM17) involving 14 immune cell states, NK cells, CAFs and endothelial cells, which contains 1351 signature genes (Table S2, Fig. S3A, and Methods). Then, we validated the ability of these gene signatures in MM17 for accurate deconvolution. We inputted it to CIBERSORT [11] to deconvolve microenvironment cell states from the artificial bulk expression profile for each patient, which was calculated as the average over all single cells in that patient from the Tirosh et al*.* dataset used in our study. Here, we calculated cell fractions of T_CD8_Mixed state and B_Non-regulatory state by aggregating those of their corresponding phenotypes (four for T and three for B cells, Methods). Notably, comparing with the actual cell state fractions, which were calculated as the relative ratios among all states by counting their corresponding cells, our predictions showed significant correlation across all tumors (*r* = 0.91, Fig. 2A). Indeed, deconvolution results were also significantly correlated for each specific cell state (*r* = 0.67 ~ 0.94, Fig. S3B) and for each individual patient (*r* = 0.61 ~ 1, Fig. S3C). Moreover, prediction results based on alternative gene signatures (LM22, supp.3, supp.12, Merged, LM22 + MM17, Merged + MM17, Methods) were less consistent than MM17, both in overall level and individual state levels (Fig. 2B), suggesting that the reference profile construction need context- and cell state-specific information. Finally, due to lack of available dataset on experimentally estimated abundances of microenvironment cell states, we attempted to validate MM17 in an external single-cell RNA-seq dataset (Jerby-Arnon et al*.* dataset) containing 2021 TME cells from 17 melanoma samples [17]. We trained a high performance classifier (accuracy = 0.87, Fig. S3D, Methods) using support vector machine (SVM) algorithm based on the Tirosh et al*.* dataset, and applied it to the Jerby-Arnon et al*.* dataset to assign each cell a cell state label. Similarly, comparing to the actual cell state proportions, deconvolution results based on MM17 showed significant concordance (*r* = 0.71, Fig. 2C). Taken together, these results demonstrated the accuracy and feasibility of MM17 on microenvironment cell states deconvolution.

### The distribution of microenvironment cell states in bulk melanoma

**Fig. 3:**
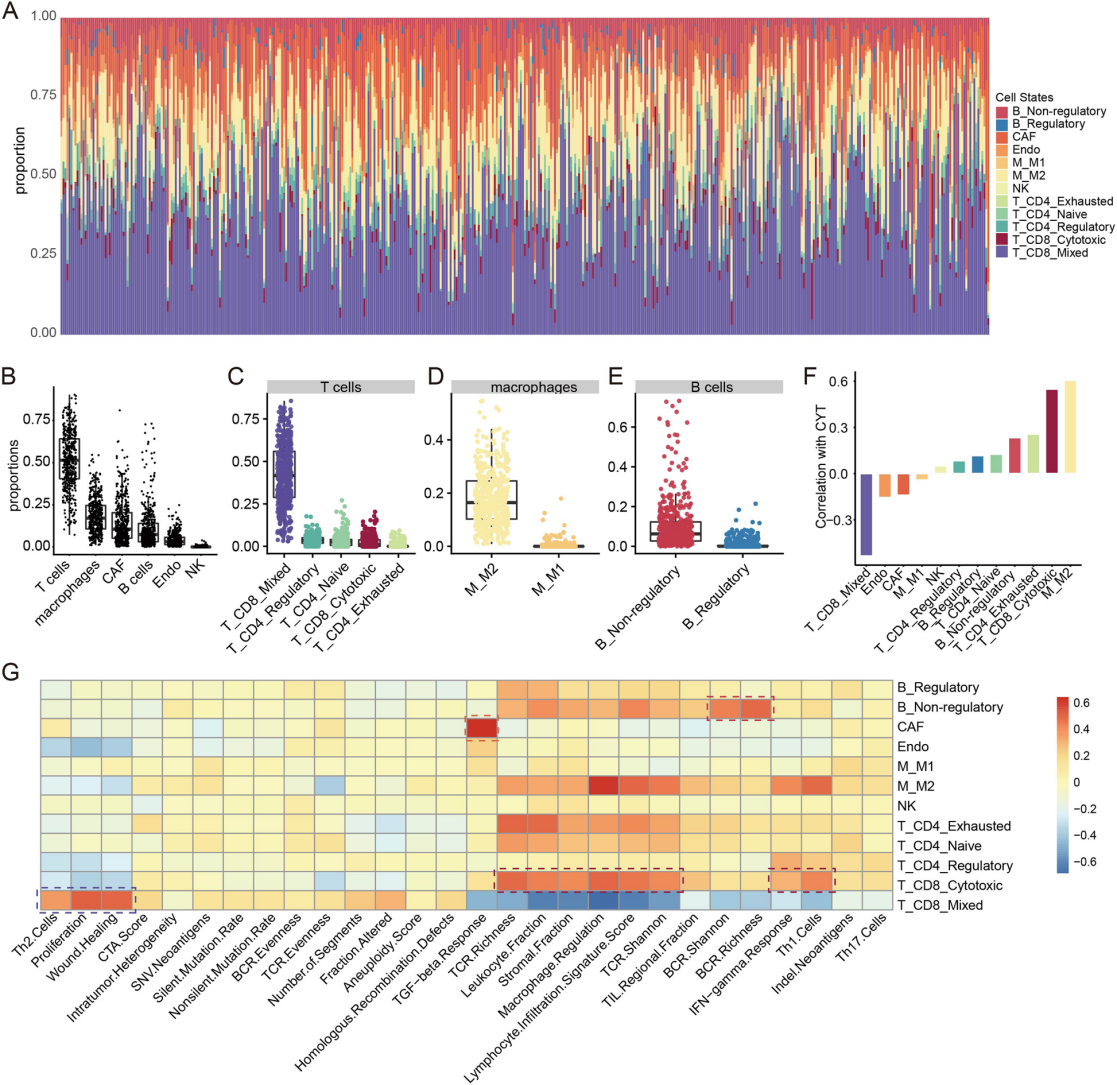
Overview of tumor microenvironment (TME) cell states in melanoma samples from TCGA. **A** Stacked bar charts summarising proportion of each TME cell state in each sample. **B**-**E** Dot plots showing the population frequency for each melanoma sample among all TME cell types (B) and among cell states in T cells (**C**), in macrophages (**D**) and in B cells (**E**). **F** Bar plots showing associations between cell states and cytolytic activity. **G** Heatmap showing associations between cell states (row) and immune characteristics (column), red for positive correlations and blue for negative correlations. Pearson's correlations were calculated. Dark red boxes highlight the strong positive associations with immune characteristics for the cell states: T_CD8_Cytotoxic, T_CD8_Mixed (Cytotoxic and Exhausted), B_Non-regulatory and CAF

To explore the landscape of microenvironment cell states in real bulk samples, we applied MM17 to 472 melanoma bulk tumors in TCGA. In total, there were 463 tumors with significant CIBERSORT *p* values (*p* value < 0.05, Fig. 3A). We observed different cellular fractions among the cell states, which displayed high variability across and within samples. Among the infiltrating microenvironment cells, T cells were the main population with a mean of 51.7% across patients. The mean frequencies of macrophages, CAFs, B cells and endothelial cells were 18.5%, 14.3%, 11.2% and 4.2%, respectively. Natural killer cells were present at very low levels with a mean of 0.1% (Fig. 3B). Among T cells, T_CD8_Mixed was the major state, while T_CD8_Cytotoxic state accounted for a small part with largest proportion up to 20.3% in melanoma patients (Fig. 3C). M_M2 state accounted for the major part of macrophage cells, which was consistent with the observation of Thorsson et al. [21] (Fig. 3D). As for B cells, a small part were with B_Regulatory state (Fig. 3E).

To investigate biological significance underlying the landscape of microenvironment cell states, we first tested for associations between fractions of individual cellular states and cytolytic activity measured based on the geometric mean of GZMA and PRF1 expression [32]. As expected, we observed strong positive correlation between T_CD8_Cytotoxic state and cytolytic activity, while T_CD8_Mixed state showed strong negative correlation with cytolytic activity (Fig. 3F). Moreover, through interrogating immune characteristics obtained from Thorsson et al*.* [21], we observed strong positive association of T_CD8_Cytotoxic fraction with lymphocyte infiltration signature score, IFN-gamma response, Th1 cells and TCR diversity (Fig. 3G), which is consistent with its functional role of eliciting antitumor immunity. While T_CD8_Mixed state was strongly correlated with tumor proliferation, wound healing and immunosuppressive Th2 cells (Fig. 3G), the latter two have been linked to poor prognosis [21, 33], suggesting its role in repressing antitumor immunity. In addition, we observed strong positive associations between CAF and TGF-beta response, and between B_Non-regulatory state and BCR diversity (Fig. 3G), these two signatures have been respectively linked to a suppressive immune response and a robust anti-tumor response [21, 34]. Finally, we confirmed pro-immunity roles of T_CD8_Cytotoxic and B_Non-regulatory states, and the roles in failure of antitumor immunity of T_CD8_Mixed state and CAF based on gene set enrichment analysis [35] (Fig. S4A-D). For instance, genes positively correlated with T_CD8_Cytotoxic fraction were enriched with lymphocyte activation, cell–cell adhesion, interferon gamma mediated signaling pathway, cell chemotaxis and so on, while genes negatively correlated were enriched with tumorigenic functions such as DNA replication, DNA repair and cell cycle (Fig. S4A). Altogether, these observations demonstrated the distribution heterogeneity of diverse cell states within individual or across SKCM tumors and their potential functional significance, supporting the strategy on microenvironment cell states deconvolution based on single-cell data.

### Association between cell states and clinico-pathological variables

Next, we investigated the association between different cell states and the standard clinico-pathological variables (Table S3), including age, gender, race, AJCC tumor stage and tumor status. After removing 3 patients each with multiple sampling, we used the remaining 457 samples to which the patients were uniquely matched for analysis. A comparison between 95 primary and 362 metastatic tumors revealed no changes of T_CD8_Cytotoxic state, while a significant decrease of M_M1 (Wilcoxon rank test *p* value = 5.6e-07) and increase of M_M2 state (Wilcoxon rank test *p* value = 9.59e-07) at metastasis (Fig. S5A), which are consistent with their anti-tumor activity of killing tumor cells and pro-tumor function of favouring tumor growth, invasion and metastasis in most cancers, respectively [8, 36].

Owing to the observed differences between primary and metastatic samples, we then focused on the 362 metastatic samples to avoid the confounding factor of the sample type and demonstrated the statistically significant associations between cell states and other clinico-pathological variables (Fig. S5). The comparison between the females and males revealed a lower frequency of CAF (Wilcoxon rank test *p* value = 0.0312), but a higher frequency of T_CD8_Cytotoxic (Wilcoxon rank test *p* value = 0.0002) cells in females (Fig. S5A). Genomic classification based on the pattern of the most prevalent significantly mutated genes identified four distinct subtypes of melanoma, including mutant BRAF (*n* = 114), mutant RAS (mainly NRAS, *n* = 81), mutant NF1(*n* = 23), and Triple-WT (wild-type, *n* = 36) [37]. Among the cell states we examined, we observed a significant increase of CAF (Mood’s test *p* value < 0.05, Wilcoxon rank test *p* value = 0.0060) and a decrease of T_CD8_Mixed (Mood’s test *p* value < 0.05, Wilcoxon rank test *p* value = 0.0004) in BRAF_Hotspot_Mutants relative to RAS_Hotspot_Mutants (Fig. S5C). BRAF and NRAS are all previously described melanoma oncogenes, indicating these cell states may contribute to the carcinogenesis. Finally, in terms of tumor status, we found a higher frequency of T_CD8_Mixed (Wilcoxon rank test p value = 6.10e-03), CAF (Wilcoxon rank test *p* value = 3.26e-05) and Endo (Wilcoxon rank test *p* value = 6.08e-03), while a lower frequency of B_Non-regulatory (Wilcoxon rank test *p* value = 1.56e-05) and T_CD8_Cytotoxic (Wilcoxon rank test *p* value = 2.45e-02) in samples with tumor status of “with tumor” compared with that of “tumor free” (Fig. S5A), indicating that patients with higher T_CD8_Mixed, CAF and lower B_Non-regulatory, T_CD8_Cytotoxic in the metastatic melanomas tend to have a higher risk of tumor recurrence during the follow-up period. These findings together demonstrated the strong association between individual cell states and clinical characteristics.

### Microenvironment cell state patterns depict distinct immune phenotypes

**Fig. 4:**
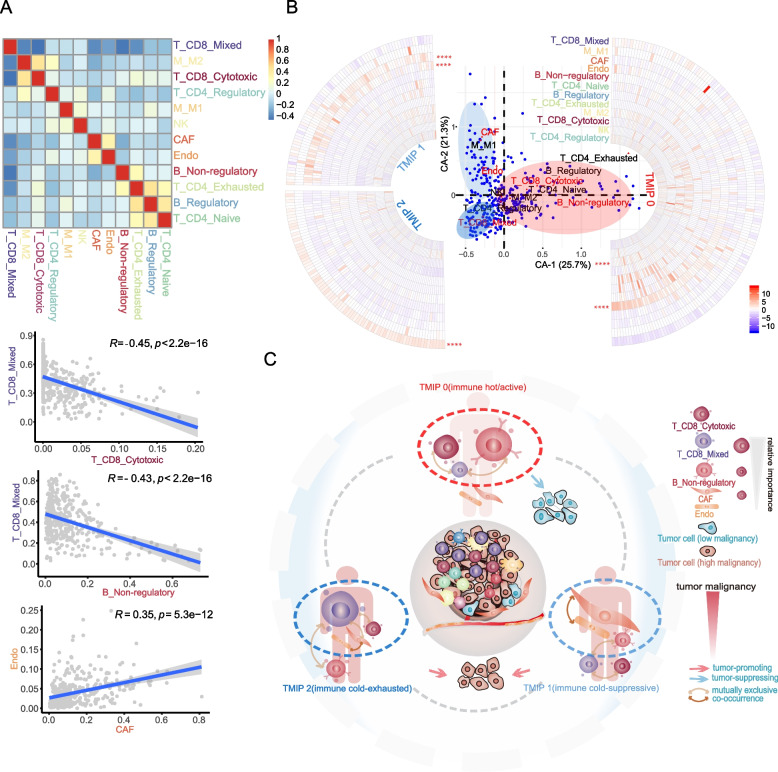
Identification of tumor-immune phenotypes. **A** Heatmap showing Pearson coefficients of correlation for relationships between each pair of cell states (top), red for positive correlations and blue for negative correlations. The cell states are colored according to cell states shown in Fig. 3A. Scatterplots showing relationships between T_CD8_Mixed (Cytotoxic and Exhausted) with T_CD8_Cytotoxic (top) and B_Non-regulatory (middle), CAF and Endo (bottom). Pearson correlations and p values are indicated. For significant correlations, linear models are shown as blue lines. **B** The first two components of correspondence analysis, accounting for 47% of the co-association structure between cell subsets and patients in the cohort, are shown. Cell states are displayed as red triangles, and patients as blue circles. Red texts highlight the important cell states for the first two components. Red, light blue and blue ellipses indicate the three patient groups of TMIP0, TMIP1 and TMIP2 classified by median values of CA-1 and CA-2, respectively. Circular heatmaps showing the z-score normalized cellular fractions for cell states (row, colored according to cell states shown in Fig. 3A) across the three patient groups (TMIP0, TMIP1 and TMIP2) classified by median values of CA-1 and CA-2. Median value difference of cell state fraction among groups was evaluated using Mood’s test. **** *P* < 0.0001 **C** The different structures of main cell states in the tumor microenvironment

To obtain a global understanding of all the microenvironment cell states, we attempted to explore their exact structures, i.e. distinct combinatorial patterns, in patients. We first quantified relationships between cell states present in the TME by simple correlation analyses. Multiple relationships were identified (Fig. 4A and S6A). Relationships among the CD8_Mixed T lymphocytes, the IL10- B lymphocytes, and the CD8_Cytotoxic T lymphocytes were observed. Samples with low levels of T_CD8_Mixed contained high amounts of the B_Non-regulatory (IL10-) and T_CD8_Cytotoxic states (*r* = -0.43 and -0.45). M_M2 was associated with T_CD8_Cytotoxic (*r* = 0.48) and CAF was associated with Endo (*r* = 0.35). In addition, B_Regulatory was associated with T_CD4_Exhausted status (*r* = 0.47).

Given the associations between different cell states, we performed correspondence analysis (CA) on the estimated cellular fractions of 316 metastatic melanoma samples with the age, gender, tumor stage and survival data all available, to obtain a smaller set of “explanatory” components. In our case, data on each patient and cell states is displayed in the same space by visualizations based on the first two components, which accounted for 47% of the co-association structure between cell subsets and patients in the cohort (Fig. 4B). Patients are organized by their tendency to contain certain cell subsets, and cell subsets are organized by their tendency to co-occur in the same patients. The most important cell states for CA-1 are the B_Non-regulatory and T_CD8_Mixed (Fig. 4B and S6B), in that patients with high CA-1 scores had higher frequencies of B_Non-regulatory, T_CD8_Cytotoxic and lower frequencies of T_CD8_Mixed, CAF (Fig. S6C and D). The second component captures a different ordering of cell states and patients, driven by mutually exclusive patterns of CAF and T_CD8_Mixed (Fig. 4B and S6B) and in that patients with high CA-2 scores had higher frequencies of CAF, Endo and lower frequencies of T_CD8_Mixed (Fig. S6C and D).

Recent studies have proposed a model in which, there are immune hot (inflamed) and cold tumors. These two phenotypes were mainly distinguished by the abundance of tumor-infiltrating lymphocytes (TILs), especially T cells with cytotoxic state [33, 38, 39]. Here, based on the median values of CA-1 and CA-2 scores in patients, we derived three tumor microenvironment immune phenotypes (TMIP) (Fig. 4B, C and Fig. S6E) that showed markedly different patterns of cell states, most notably T_CD8_Cytotoxic, T_CD8_Mixed, B_Non-regulatory and CAF, all of which were implicated in antitumor immunity as shown above. Accordingly, patients were separated into three groups with their corresponding TMIPs (*n* = 158, 77 and 81, respectively). TMIP 0 (high CA-1, “immune hot/active”) was defined by higher B_Non-regulatory, T_CD8_Cytotoxic states and a lower T_CD8_Mixed state, which displayed an active immune response underlined by the functional impact of its corresponding cell states on tumor immunity. TMIP 1 (low CA-1/high CA-2, “immune cold-suppressive”) was mainly characterized by higher CAF, Endo and a lower B_Non-regulatory, which showed a suppressive immune response dominated by CAF [40, 41]. While, the TMIP 2 (low CA-1/low CA-2, “immune cold-exhausted”) exhibited the highest T_CD8_Mixed and lowest T_CD8_Cytotoxic, CAF (Mood’s test *p* value < 0.05, Wilcoxon rank test *p* value < 0.05), indicating an exhausted immune response [42]. We named the TMIPs based on two immune characteristics: the abundance of T cells with cytotoxic state used to distinguish hot and cold tumors, and the microenvironment cell state patterns used to depict immune response types. These results revealed different patterns of cell states in the melanoma patients, which depicted distinct types of immune response (or immune activity).

### Prognostic significance of the immune phenotypes

**Fig. 5:**
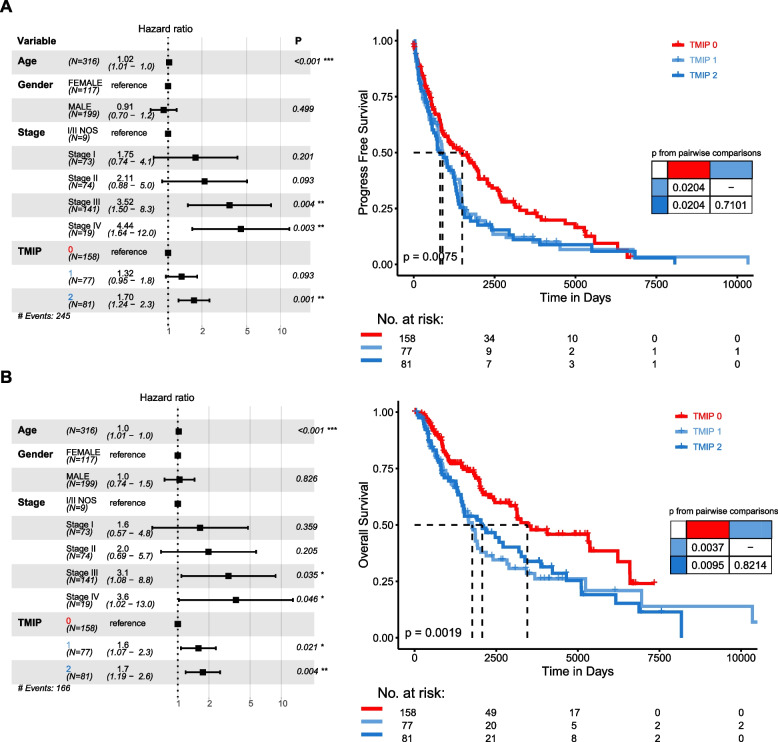
Prognostic associations of immune phenotypes. **A** Forest plot of multivariable hazard ratios for PFS (left) and Kaplan–Meier curves showing PFS of patients (right). **B** As in (**A**), prognostic associations for OS. Multivariable analysis was adjusted for age, gender and tumor stage. Depicted p values for Kaplan–Meier curves are from log-rank tests

We next investigated how the patterns of diverse cell states in the TME influence patient survival. The median follow-up of the 316 metastatic patients studied above was 4.2 years, with the type of follow-up being progress-free survival (PFS) for 245 events and overall survival (OS) for 166 events. In univariate analysis, both the TMIP 1 and TMIP 2 groups were associated with worse PFS (Tables S4 and 5) compared with the TMIP 0 (HR_1 vs 0_ = 1.4557, 95% CI = 1.0724–1.9761, *p* = 0.016; HR_2 vs 0_ = 1.537, 95% CI = 1.1305–2.0898, *p* = 0.0061). Using multivariate Cox regression adjusted for age, gender and tumor stage, we identified some significant associations between these TMIPs and patient outcomes (Fig. 5). TMIP 1 was significantly associated with worse OS (HR_1 vs 0_ = 1.5832, 95% CI = 1.0726–2.3369, *p* = 0.0207), and showed moderately significant association with PFS (HR_1 vs 0_ = 1.3168, 95% CI = 0.955–1.8157, *p* = 0.0932). Remarkly, TMIP 2 showed a more significant association with worse PFS (HR_2 vs 0_ = 1.7003, 95% CI = 1.238–2.3354, *p* = 0.001) and OS (HR_2 vs 0_ = 1.7448, 95% CI = 1.1886–2.5615, *p* = 0.0045) (Fig. 5A and B). These results revealed the potential prognostic implications of our identified immune phenotypes for melanoma patients.

### Implications of immune phenotypes in response to immune checkpoint blockade therapy

**Fig. 6:**
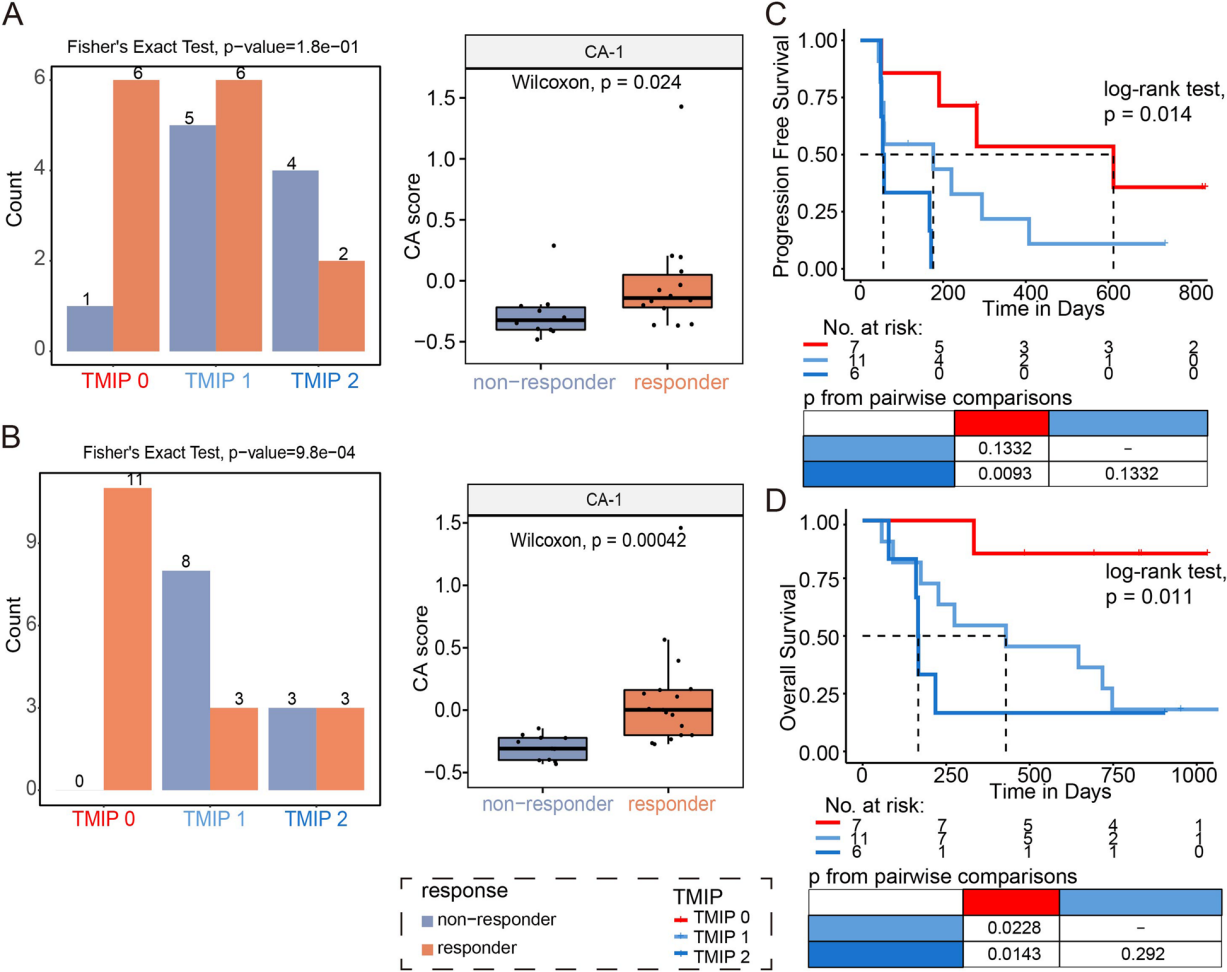
Association between tumor microenvironment immune phenotypes (TMIP) and response to immune checkpoint blockade (ICB) in melanoma. **A**-**B** Bar charts showing numbers of responders and non-responders with different TMIPs in anti-PD1 pre-treatment patients (**A**) and on-treatment patients (**B**) who progressed after a first-line anti-CTLA4 treatment (Ipi-progressed) in Riaz et al*.* data. Box plots showing differences of CA-1 scores between responders and non-responders in those corresponding patients. **C**-**D** Kaplan–Meier curves showing differences of progression-free survival (**C**) and overall survival (**D**) among patients with different TMIPs in Ipi-progressed patients in Riaz et al*.* data

Cancer cells exploit multiple mechanisms in order to avoid the immune attack, fortunately, immunotherapy strategy with checkpoint blockade has been raised as a promising weapon against immune escape. Here, having showed the prognostic significance of immune phenotypes, we attempted to explore the relation of immune phenotypes to checkpoint therapy response. We first focused on 36 pre-treatment SKCM samples with available information on response to immunotherapy, in which 21 samples had response while 15 had no response. Patients with TMIP 0 showed a higher proportion of responders comparing to patients with TMIP2 (Fig. S7A, 68.4% for TMIP 0 and 36.4% for TMIP 2). Then, we utilized a recently generated RNA-seq profiles of melanoma patients treated with anti-PD1 containing pre-treatment and on-treatment (i.e. during therapy) patients (Table S6) [25]. We predicted CA-1 and CA-2 scores used to identify immune phenotypes of these samples based on the model constructed with TCGA data, and classified them into the three TMIP groups based on the corresponding thresholds of CA-1 and CA-2 scores obtained from TCGA data (Fig. S7B and Methods). Among the 24 anti-PD1 pre-treatment tumors which progressed after a first-line anti-CTLA4 treatment (Ipi-progressed), CA-1 scores showed significantly higher levels in 14 responders than in 10 non-responders (Fig. 6A, Wilcoxon rank test, *p* value = 0.024). And, we observed that cell proportions of B_Non-regulatory showed significant increase in responders compared to non-responders (Fig. S7D). Accordingly, tumors with TMIP 0 (high CA-1) had a 85.7% response, compared to 54.5% for those with TMIP1 and 33.3% for TMIP 2 (Fig. 6A, Fisher’s exact test, *p* value = 0.1 for TMIP0 and TMIP2). Moreover, patients with TMIP 0 showed significantly better OS than those with TMIP 1, and patients with TMIP 2 showed worse PFS and OS (Fig. 6C-D, log-rank *p* values = 0.014 and 0.011 for PFS and OS, respectively). Notably, when using transcriptomic profiles of 28 on-treatment patients, Ipi-progressed ones with TMIP 0 were all responders and those with TMIP 1 and TMIP 2 contained several non-responders (Fig. 6B, 100% for TMIP 0, 27.3% for TMIP 1 and 50% for TMIP 2, Fisher’s exact test, *p* value = 9.8e-4). The comparison between the responders and non-responders revealed a significantly higher frequency of both B_Non-regulatory and T_CD8_Cytotoxic (Wilcoxon rank test *p* value < 0.05) cells in responders (Fig. S7E). Our results demonstrated that the progressed patients with more B_Non-regulatory, T_CD8_Cytotoxic and a less T_CD8_Mixed state (TMIP 0, “immune hot/active”) tended to response to anti-PD1 therapy, compared to the “immune cold-suppressive” (TMIP 1) or “immune cold-exhausted” (TMIP 2) phenotypes.

## Discussion

The extensive studies on immune system function in tumor development underline successful immunotherapy in diverse cancer types [43, 44]. Although immunologic ignorance and active inhibition can cause tumor escape, immune cells can still mediate tumor development [45]. The heterogeneous composition of microenvironment cells and their states in tumors remain studied to further understand the mechanisms of immune therapy. Leveraging reference profiles of microenvironment cell states that were constructed based on single-cell RNA-seq data, we performed cell state-based deconvolution of gene expression profiles and mapped the landscape of microenvironment cell states across melanoma bulk tumors from TCGA. Then, by exploring the patterns of these cell states in TME, we identified three tumor microenvironment immune phenotypes: “immune hot/active”, “immune cold-suppressive” and “immune cold-exhausted”, and revealed their associations with patient survival and response to immune checkpoint therapy in an independent cohort of checkpoint-treated melanoma patients.

In this study, we captured the primary functional status that play important roles in antitumor immunity through the analysis of ~ 2700 immune cells of T, B lymphocytes and macrophages. The exhaustion state of T cells is usually depicted by dynamic transformation from effector functions, and persistent expression of diverse inhibitory receptors. The strength of exhaustion is mediated by many factors, including the level and count of expressed inhibitory molecules and the intensity of antigen stimulation [46]. Several studies have revealed intermediate states between effector and exhausted CD8 + T cells in cancers [26, 47] and a hierarchy of CD8 + T cell exhaustion in human melanoma [28]. Here, we found that a mixed state of T_CD8_Cytotoxic-Exhausted with its phenotypic diversity existed in melanoma, indicating the presence of possible early or partially exhausted T cells. Since T cells transition to new states in cancer or chronic infections [48, 49], we used a nonlinear dimensionality-reduction technique UMAP [50] to visualize the intercluster relationships of T cells (Fig. S1A), which showed some overlap. The observation of the proximity between subclusters of T_CD8_cytotoxic state (C3) and T_CD8_Mixed state (C1, C2 and C4) suggests that these diverse phenotypes of CD8 + T cells with mixed state may arise from the cytotoxic state. By contrast, the role of B cells in solid tumor development is not established and they were mainly studied in B cell malignancies (e.g. chronic lymphocytic leukemia) [51]. In our study, we found a subset of B cells with regulatory phenotype and the vast majority non-regulatory. While all cell types, subtypes and states cannot possibly be described here in full, we expect that as more and more scRNA-seq data with large microenvironment cells of the same tumor types available, future studies will need to integrate these single-cell transcriptomic data to determine the more subtle states of individual cells.

ScRNA-seq provides high-dimensional, single-cell level data, yet the difficulty in obtaining fresh tumor tissue and other technical limitations imply that it currently is not yet suitable for studies of large patient cohorts. Conventionally, most studies utilized the normal leukocyte reference profile LM22 to dissect cellular composition of tumor microenvironment from bulk tumors [11, 52, 53], which differed from those of TME substantially, and thus could lead to less accurate results [14]. Here, combining reference profiles of microenvironment cell states with the robust performance of CIBERSORT allowed us to accurately estimate the abundance for different cell states as a fraction of the overall cells we studied. We successfully mapped the cellular states of infiltrating microenvironment cells (e.g. T_CD8_Cytotoxic and T_CD8_Mixed states) in the SKCM samples, and revealed their distribution heterogeneity both across and within samples. Especially, T_CD8_Mixed sate largely existed across SKCM samples, highlighting its immunosuppressive role to promote carcinogenesis and progression. Importantly, by analysis of the structures (i.e. combinatorial patterns) of cell states, we identified three tumor microenvironment immune phenotypes (TMIP) in SKCM samples: “immune hot/active”, “immune cold-suppressive” and “immune cold-exhausted”. These phenotypes were characterized by distinct structures of cell states, most notably T_CD8_Cytotoxic, T_CD8_Mixed, B_Non-regulatory and CAF, which depicted distinct types of antitumor immune response (or immune activity). Our results suggest that the abundance of individual cell state is not sufficient to delineate the tumor-immune phenotype, and that it is important to also consider other cell states in the TME which collectively affect tumor immunity. To some extent, our study on the microenvironment cell states could help highlighting their importance in tumor biology and understanding heterogeneous response to immunotherapy.

Finally, we revealed associations of the immune phenotype with melanoma survival and immunotherapeutic benefits. The “immune hot-active” showed a positive correlation with progression-free survival, while patients with “immune cold-suppressive” or “immune cold-exhausted” phenotypes had shorter progression-free survival. We further found its association with response to immunotherapy in an independent cohort of checkpoint-treated (anti-PD1) melanoma patients [25] with explicit information on therapeutic drugs and outcomes. We observed a higher proportion of responders in patients with “immune hot/active” phenotype when compared to those with “immune cold-exhausted” in pre-treatment tumors, which progressed after a first-line anti-CTLA4 treatment (Ipi-progressed). Moreover, patients with “immune hot/active” phenotype showed better OS than those with “immune cold-suppressive” phenotype, and patients with “immune cold-exhausted” phenotype had worse prognosis (PFS and OS). Notably, among that of on-treatment patients, we also observed a higher proportion of responders in patients with “immune hot/active” when compared to those with “immune cold-suppressive” or “immune cold-exhausted”. These results together pointed to the less favorable microenvironment of “suppressive” or “exhausted” phenotypes for immunotherapy, compared to that of “immune hot/active”. Although there were no obvious difference between “suppressive” and “exhausted” phenotypes on clinical outcome, the different cellular states between them could provide therapy basis in further study. Complementarily, Jiang et al. recently proposed the TIDE signature which could predict cancer immunotherapy response, and their results showed good prediction performance among Ipi-naive patients while no efficiency among Ipi-progressed patients using pre-treatment tumors [54]. Thus, it is possible that combining TIDE with TMIP could show more robust performance in prediction of immunotherapy response. Several other biomarkers have also been proposed, such as tumor neoantigen load [55], and signatures of mesenchymal transition, wound healing, and angiogenesis [56]. As limited efficiency was achieved due to the complex interplay between tumor genomics, epigenomics and anti-tumor immune responses, we anticipate better prediction performance through further incorporating multiple biomarkers with additional information on immune infiltrates such as spatial distribution [57].

## Conclusions

In summary, leveraging single-cell RNA-seq data, we dissected the composition of microenvironment cell states in melanoma tumors at bulk level and revealed different immune phenotypes characterized by distinct cell states, highlighting the importance of microenvironment cell states for the understanding of tumor immunity in individual tumors and providing a new strategy for patient stratification.

### Supplementary Information

**Additional file 1: Figure S1. **Immune cell expression heterogeneity and cell subsets distribution across patients, related to Fig. 1. (A) UMAP projection of 2068 single T cells (left), 515 B cells (middle) and 126 macrophages (right) from 19 patients. Each dot corresponds to one single cell, colored according to cell cluster. (B) Heatmap of T cell clusters (left), B cell clusters (middle) and macrophage clusters (right) with unique signature genes. Top 20 specifically expressed genes are marked alongside, if available. (C-E) Bar plots showing the number (left panel) and fraction (right panel) of cells originating from the 19 patients for each subcluster of T cells (C), B cells (D) and macrophages (E). (F) The fractions of the 15 subclusters, NK cells, CAFs and endothelial cells in each patient. **Figure S2. **Cell subcluster characterization of functional status. (A) Top 100 ranked (based on fold change) differentially expressed genes indicative of the functional status in each T-cell cluster (top) and z-score normalized mean expression of known functional marker sets across single T cells (bottom). The numbers in parentheses correspond to the ranks and the key markers (Table S1) are highlighted by red color. (B) Heatmap showing the log2-transformed expression of selected T cell function-associated genes in single cells. (C) Violin plots showing the expression profile of selected genes involved in T-cell cytotoxicity (top) and exhaustion (bottom), stratified by T-cell clusters. (D) Top 100 ranked (based on fold change) differentially expressed genes indicative of the functional status in each cluster (C1, C2 and C3 for B cells; C0 and C1 for macrophages). The numbers in parentheses correspond to the ranks and the key markers (Table S1) are highlighted by red color. (E) Z-score normalized mean expression of known functional marker sets across single B cells (top) and the log2-transformed expression of selected B cell function-associated genes in single cells (bottom). (F-G) Heatmaps showing the z-score normalized mean expression of known functional marker sets across single macrophages and their log2-transformed expression in single cells. Blue boxes highlight the key markers and the numbers in brackets represent the total times appeared in literature. **Figure S3.** MM17 reference profile and performance assessment. (A) Heatmap of MM17 reference profile depicting z-score normalized expression of each gene across 17 tumor microenvironment (TME) cell subsets. (B-C) Correlation between predicted proportions and true proportions for each individual cell state (B) and for each individual patient (C). (D) Confusion matrix of all TME cell states. **Figure S4. **Functional associations of tumor microenvironment (TME) cell states. (A-D) Enriched GO biological processes of T_CD8_Cytotoxic (A), B_Non-regulatory (B), T_CD8_Mixed (C) and CAF (D) based on gene set enrichment analysis (GSEA). **Figure S5. **Associations between cell states and clinico-pathological variables. (A-C) Associations of molecular and clinical features with cell states. (A) Boxplots showing the cell fraction distribution of each cell state stratified by tumor type (left), gender (middle) and tumor status (right). (B) Boxplots showing the cell fraction distribution of each cell state stratified by integrative age (left), tumor stage (middle), and race (right). (C) The fraction distribution of cell states stratified by TCGA subtypes. Median value difference of cell fraction among subtypes was evaluated using Mood’s test. Wilcoxon rank sum tests were used to examine the significance of the differences between two groups. For tumor stage, patients with Stage 0, Stage I, IA, IB, Stage II, IIA, IIB and IIC are grouped as “LOW” (*n*=154), Stage III, IIIA, IIIB, IIIC and Stage IV are grouped as “HIGH” (*n*=162). * *P* < 0.05, ** *P* < 0.01, *** *P* < 0.001, **** *P* < 0.0001. **Figure S6.** Associations between cell states and immune phenotypes, related to Fig. 4. (A) Scatterplots showing relationships between T_CD8_Cytotoxic and M_M2 (top), B_Regulatory and T_CD4_Exhausted (middle), CAF and T_CD8_Mixed (Cytotoxic and Exhausted) (bottom). Pearson correlations and *p* values are indicated. For significant correlations, linear models are shown as blue lines. (B) Contributions of the cell states to CA-1 (top) and CA-2 (bottom). (C) Scatter chart of the Pearson correlations of CA-1 and CA-2 with cell states. Different colors indicate whether or not significant associations between CA scores and cell states were observed (*p* < 0.05). (D) Boxplots showing the cell fraction distribution of each cell state stratified by the median values of CA-1 (top) and CA-2 (bottom), respectively. Wilcoxon rank sum tests were used to examine the significance of the differences between two groups. (E) The distribution of cell states across the three immunophenotype groups classified by median values of CA-1 and CA-2. Median value difference of cell fraction among groups was evaluated using Mood’s test. Then the statistical significance between any two groups was evaluated by Wilcoxon rank sum test and p values are shown at the top of each panel. * *P* < 0.05, ** *P* < 0.01, *** *P* < 0.001, **** *P* < 0.0001. **Figure S7. **Assessment on association between tumor microenvironment immune phenotypes (TMIP) and response to immune checkpoint blockade (ICB) in melanoma. (A) Box plots showing differences of CA-1 (upper panel) and CA-2 (middle panel) scores between responders and non-responders in patients under immunotherapy in TCGA data. Bar charts showing numbers of responders and non-responders with different TMIPs in those patients (lower panel). (B) Projection of each patient of Riaz *et al.* dataset onto the first and second component of the correspondence analysis. Left panel showed pre-treatment samples and right panel denoted on-treatment patients. Non-responders were colored blue, and responders were colored orange. Points denoted Ipi-naive patients, and triangles denoted Ipi-progressed patients. (C) Box plots showing differences of CA-2 scores between responders and non-responders in anti-PD1 pre-treatment patients (upper panel) and on-treatment patients (lower panel) who progressed after a first-line anti-CTLA4 treatment (Ipi-progressed) in Riaz *et al. *data. (D-E) Comparison of each cell state proportion between responders and non-responders in Ipi-progressed patients based on pre-treatment (D) and on-treatment (E) transcriptomic profiles. ns: not significant; *: *p* < 0.05. **Table S1.** Gene lists used for functional analyses. **Table S3.** Demographics and characteristics of patients with melanoma. **Table S4. **Uni- and multivariate analysis for progress-free survival (316 sample). **Table S5.** Uni- and multivariate analysis for overall survival (316 sample).

**Additional file 2: Table S2.** MM17 reference profile.

**Additional file 3: Table S6.** Summary of characteristics for Riaz et al. dataset.

## Data Availability

The datasets analysed during the current study are available either from Gene Expression Ominibus (GEO) repository (https://www.ncbi.nlm.nih.gov/geo/): GSE72056 and GSE115978, the OSFHOME (https://osf.io/cxj8h/?show=view) or from the authors upon reasonable request.
