# Supplementary material for: Dissecting cellular states of infiltrating microenvironment cells in melanoma by integrating single-cell and bulk transcriptome analysis
Source: BMC Immunol. 2023 Dec 12;24:52. doi: 10.1186/s12865-023-00587-8 (PMC10714533; doi:10.1186/s12865-023-00587-8)
Supplement: Supplementary file 1 — Additional file 1: Figure S1. Immune cell expression heterogeneity and cell subsets distribution across patients, related to Fig. 1. (A) UMAP projection of 2068 single T cells (left), 515 B cells (middle) and 126 macrophages (right) from 19 patients. Each dot corresponds to one single cell, colored according to cell cluster. (B) Heatmap of T cell clusters (left), B cell clusters (middle) and macrophage clusters (right) with unique signature genes. Top 20 specifically expressed genes are marked alongside, if available. (C-E) Bar plots showing the number (left panel) and fraction (right panel) of cells originating from the 19 patients for each subcluster of T cells (C), B cells (D) and macrophages (E). (F) The fractions of the 15 subclusters, NK cells, CAFs and endothelial cells in each patient. Figure S2. Cell subcluster characterization of functional status. (A) Top 100 ranked (based on fold change) differentially expressed genes indicative of the functional status in each T-cell cluster (top) and z-score normalized mean expression of known functional marker sets across single T cells (bottom). The numbers in parentheses correspond to the ranks and the key markers (Table S1) are highlighted by red color. (B) Heatmap showing the log2-transformed expression of selected T cell function-associated genes in single cells. (C) Violin plots showing the expression profile of selected genes involved in T-cell cytotoxicity (top) and exhaustion (bottom), stratified by T-cell clusters. (D) Top 100 ranked (based on fold change) differentially expressed genes indicative of the functional status in each cluster (C1, C2 and C3 for B cells; C0 and C1 for macrophages). The numbers in parentheses correspond to the ranks and the key markers (Table S1) are highlighted by red color. (E) Z-score normalized mean expression of known functional marker sets across single B cells (top) and the log2-transformed expression of selected B cell function-associated genes in single cells (bottom). (F- [file 12865_2023_587_MOESM1_ESM.zip › Supplemental Information.docx]

**Dissecting cellular states of infiltrating microenvironment cells in melanoma by integrating single-cell and bulk transcriptome analysis**

**Aiai Shi et al.**

Supplemental Information

# Supplementary Figures and Tables

## Supplementary Figures





**Figure S1. Immune cell expression heterogeneity and cell subsets distribution across patients, related to figure 1.** (A) UMAP projection of 2068 single T cells (left), 515 B cells (middle) and 126 macrophages (right) from 19 patients. Each dot corresponds to one single cell, colored according to cell cluster. (B) Heatmap of T cell clusters (left), B cell clusters (middle) and macrophage clusters (right) with unique signature genes. Top 20 specifically expressed genes are marked alongside, if available. (C-E) Bar plots showing the number (left panel) and fraction (right panel) of cells originating from the 19 patients for each subcluster of T cells (C), B cells (D) and macrophages (E). (F) The fractions of the 15 subclusters, NK cells, CAFs and endothelial cells in each patient.


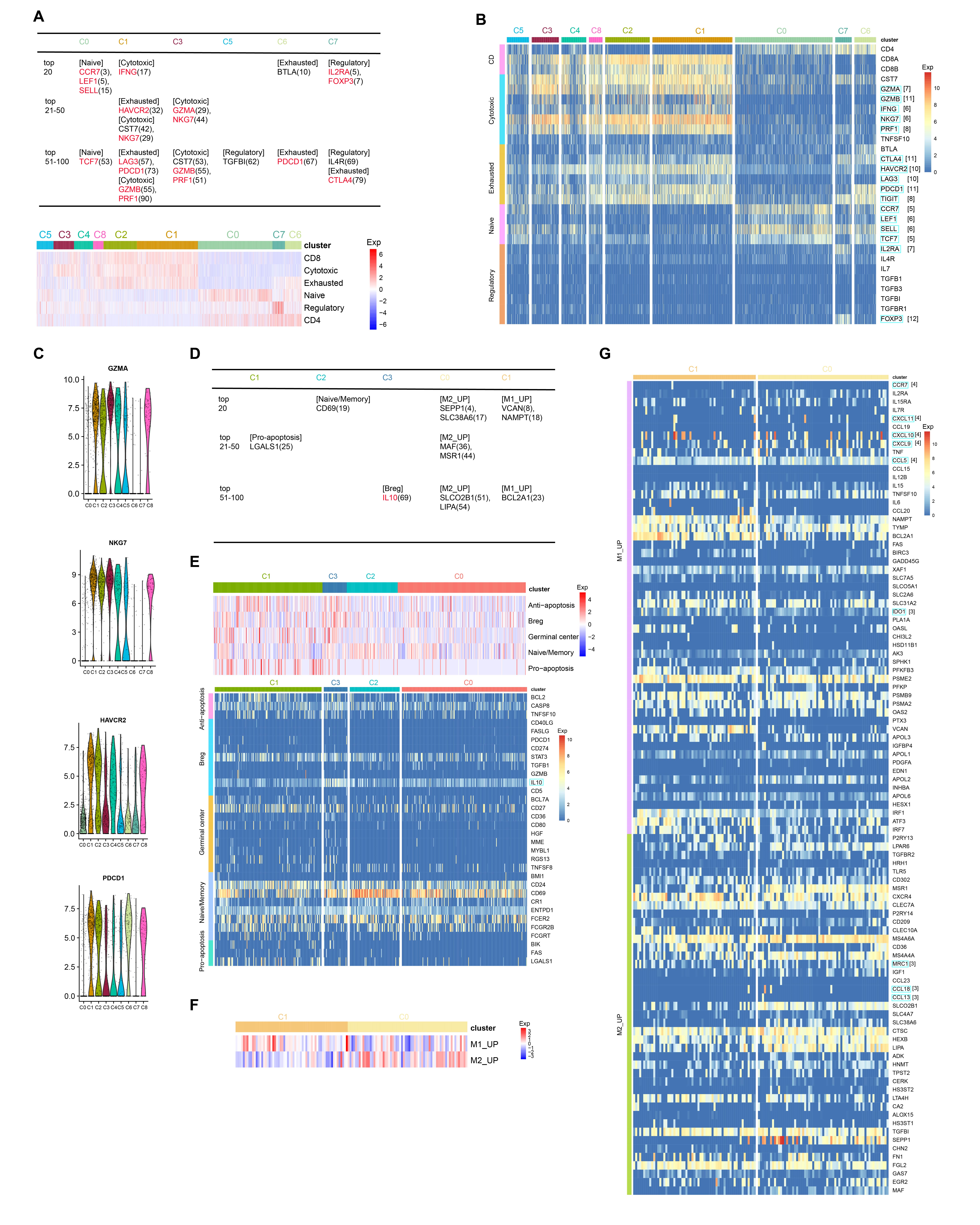


**Figure S2. Cell subcluster characterization of functional status.** (A) Top 100 ranked (based on fold change) differentially expressed genes indicative of the functional status in each T-cell cluster (top) and z-score normalized mean expression of known functional marker sets across single T cells (bottom). The numbers in parentheses correspond to the ranks and the key markers (Table S1) are highlighted by red color. (B) Heatmap showing the log2-transformed expression of selected T cell function-associated genes in single cells. (C) Violin plots showing the expression profile of selected genes involved in T-cell cytotoxicity (top) and exhaustion (bottom), stratified by T-cell clusters. (D) Top 100 ranked (based on fold change) differentially expressed genes indicative of the functional status in each cluster (C1, C2 and C3 for B cells; C0 and C1 for macrophages). The numbers in parentheses correspond to the ranks and the key markers (Table S1) are highlighted by red color. (E) Z-score normalized mean expression of known functional marker sets across single B cells (top) and the log2-transformed expression of selected B cell function-associated genes in single cells (bottom). (F-G) Heatmaps showing the z-score normalized mean expression of known functional marker sets across single macrophages and their log2-transformed expression in single cells. Blue boxes highlight the key markers and the numbers in brackets represent the total times appeared in literature.


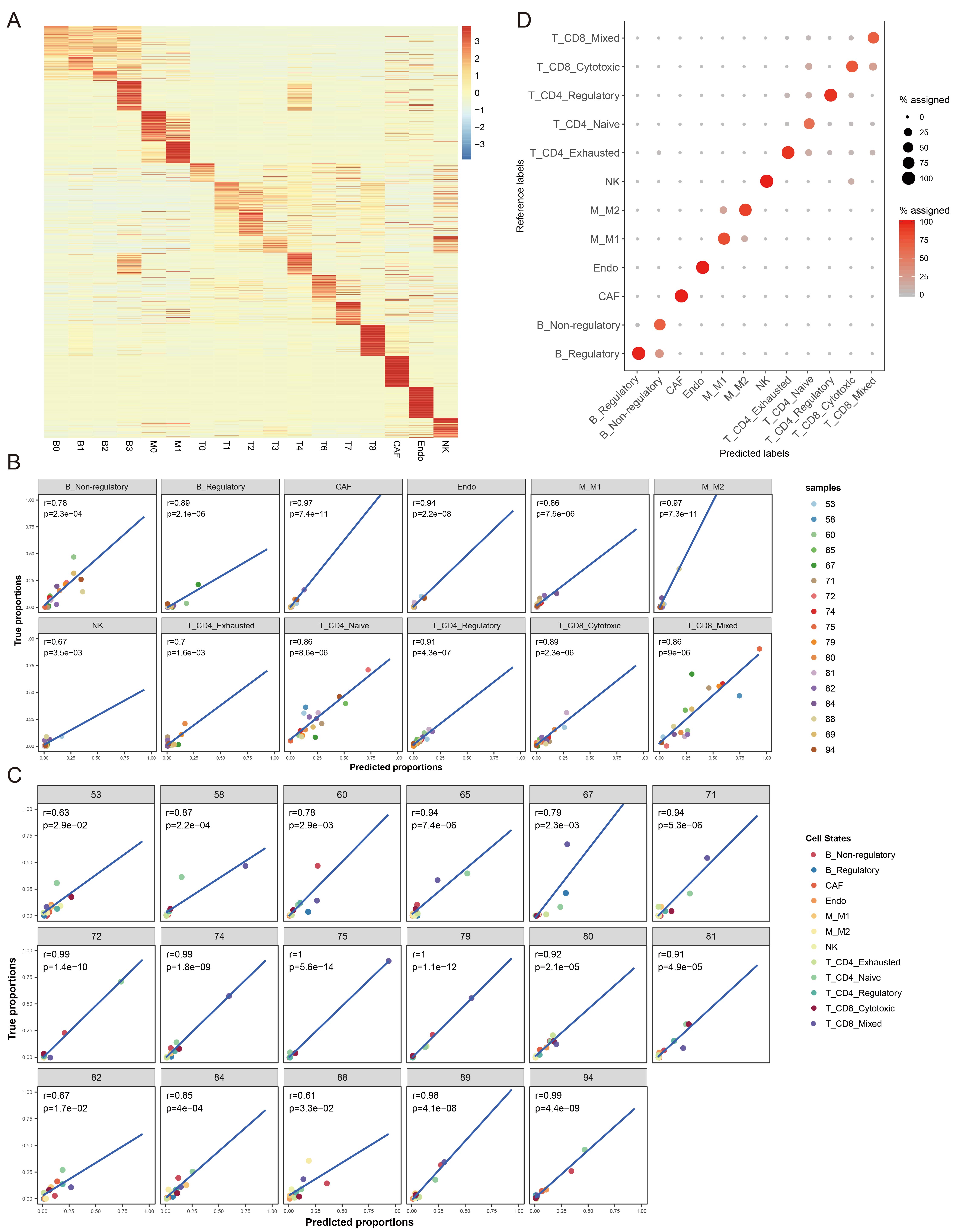


**Figure S3. MM17 reference profile and performance assessment.** (A) Heatmap of MM17 reference profile depicting z-score normalized expression of each gene across 17 tumor microenvironment (TME) cell subsets. (B-C) Correlation between predicted proportions and true proportions for each individual cell state (B) and for each individual patient (C). (D) Confusion matrix of all TME cell states.


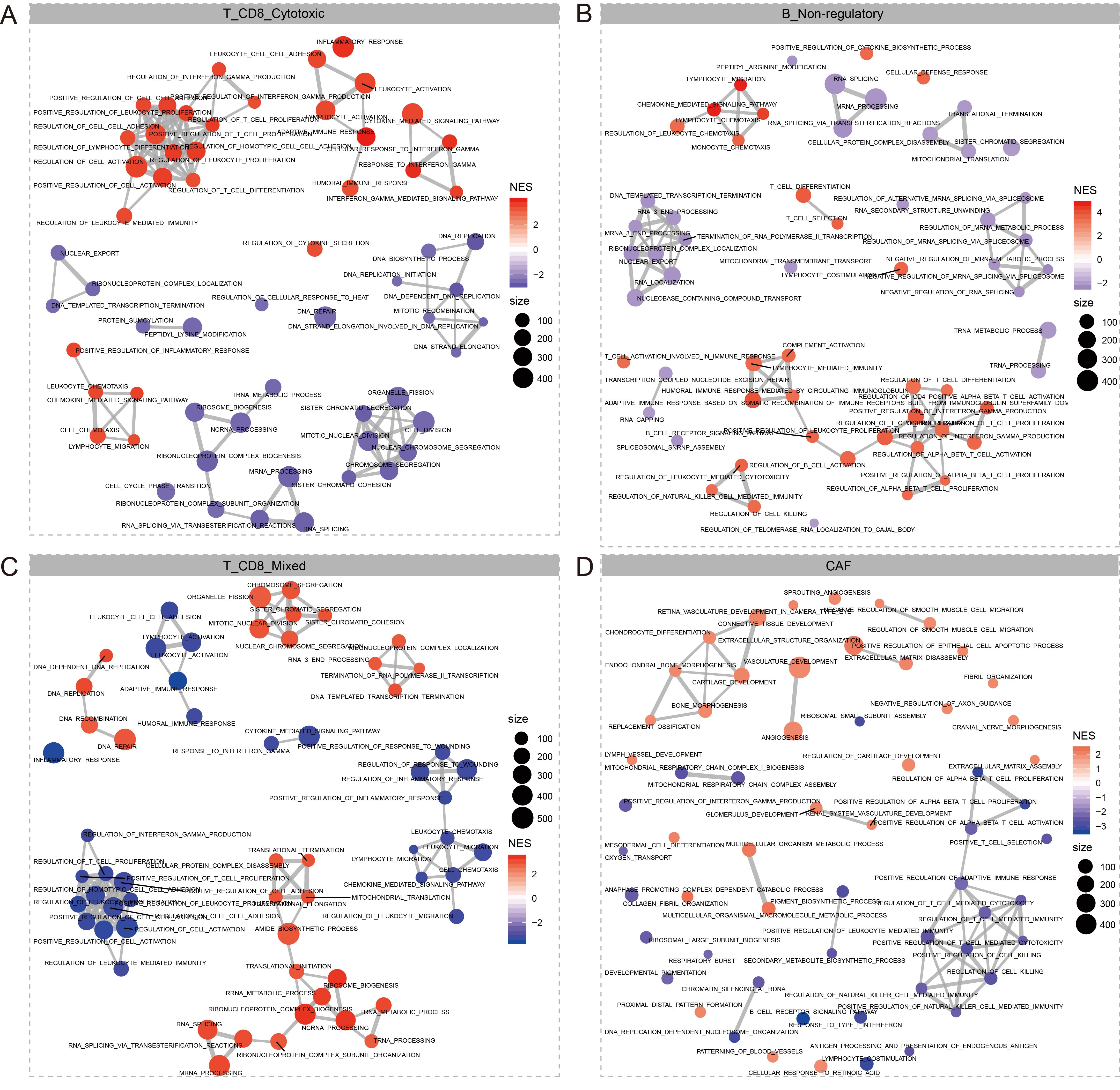


**Figure S4. Functional associations of tumor microenvironment (TME) cell states.** (A-D) Enriched GO biological processes of T_CD8_Cytotoxic (A), B_Non-regulatory (B), T_CD8_Mixed (C) and CAF (D) based on gene set enrichment analysis (GSEA).


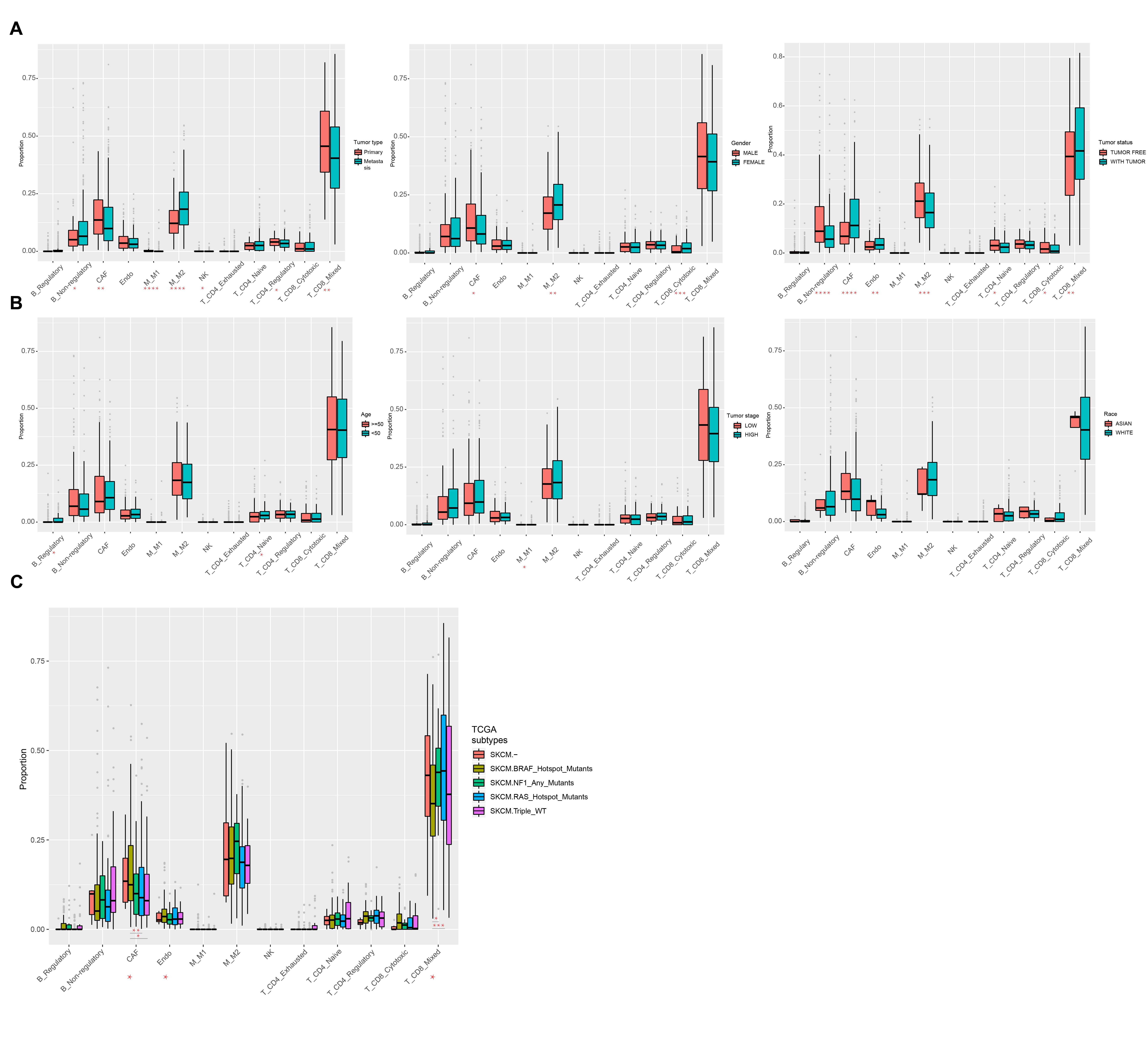


**Figure S5. Associations between cell states** **and clinico-pathological variables.** (A-C) Associations of molecular and clinical features with cell states. (A) Boxplots showing the cell fraction distribution of each cell state stratified by tumor type (left), gender (middle) and tumor status (right). (B) Boxplots showing the cell fraction distribution of each cell state stratified by integrative age (left), tumor stage (middle), and race (right). (C) The fraction distribution of cell states stratified by TCGA subtypes. Median value difference of cell fraction among subtypes was evaluated using Mood’s test. Wilcoxon rank sum tests were used to examine the significance of the differences between two groups. For tumor stage, patients with Stage 0, Stage I, IA, IB, Stage II, IIA, IIB and IIC are grouped as “LOW” (n=154), Stage III, IIIA, IIIB, IIIC and Stage IV are grouped as “HIGH” (n=162). * P < 0.05, ** P < 0.01, *** P < 0.001, **** P < 0.0001


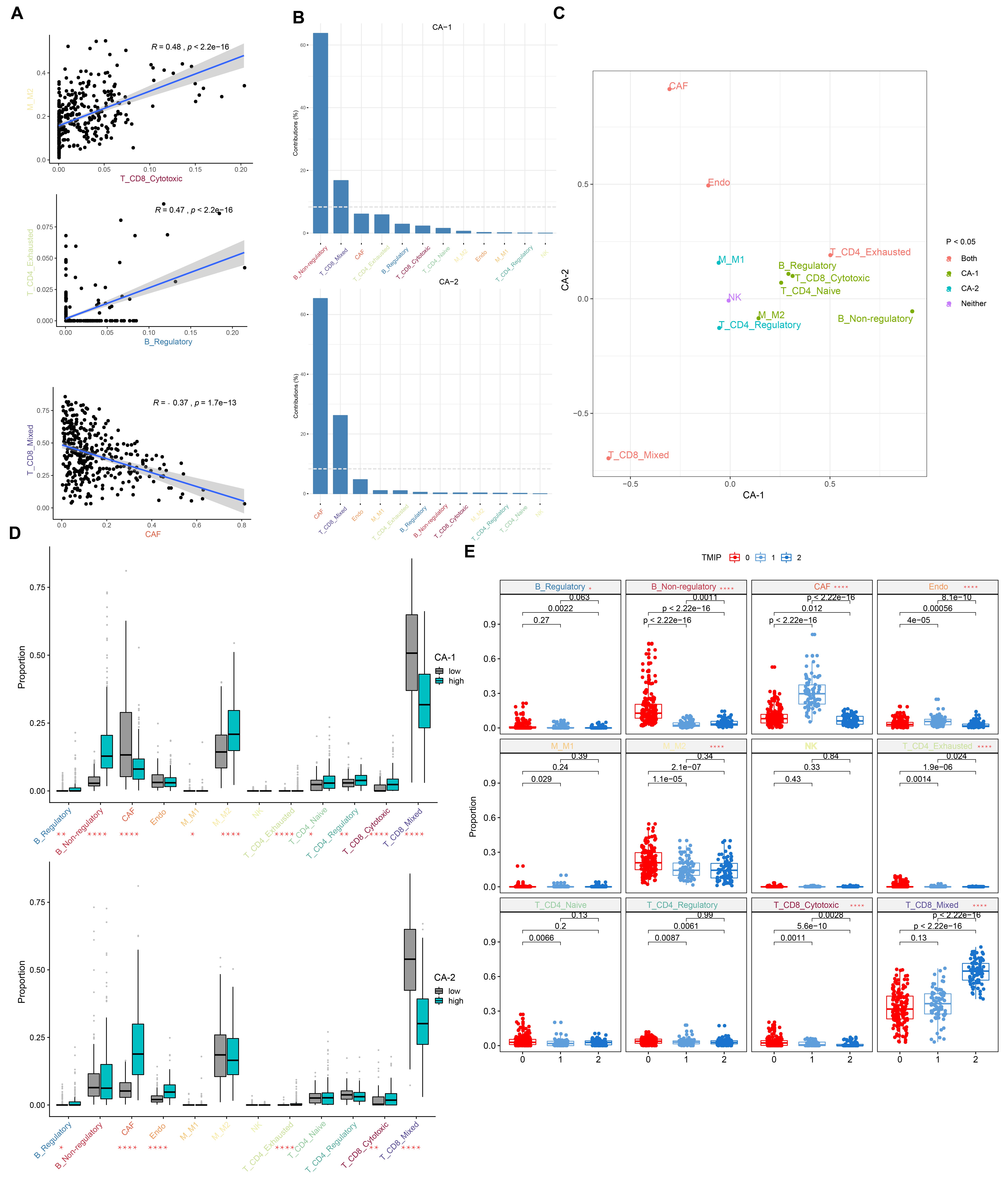


**Figure S6.** **Associations between cell states and immune phenotypes, related to figure 4.** (A) Scatterplots showing relationships between T_CD8_Cytotoxic and M_M2 (top), B_Regulatory and T_CD4_Exhausted (middle), CAF and T_CD8_Mixed (Cytotoxic and Exhausted) (bottom). Pearson correlations and p values are indicated. For significant correlations, linear models are shown as blue lines. (B) Contributions of the cell states to CA-1 (top) and CA-2 (bottom). (C) Scatter chart of the Pearson correlations of CA-1 and CA-2 with cell states. Different colors indicate whether or not significant associations between CA scores and cell states were observed (p < 0.05). (D) Boxplots showing the cell fraction distribution of each cell state stratified by the median values of CA-1 (top) and CA-2 (bottom), respectively. Wilcoxon rank sum tests were used to examine the significance of the differences between two groups. (E) The distribution of cell states across the three immunophenotype groups classified by median values of CA-1 and CA-2. Median value difference of cell fraction among groups was evaluated using Mood’s test. Then the statistical significance between any two groups was evaluated by Wilcoxon rank sum test and p values are shown at the top of each panel. * P < 0.05, ** P < 0.01, *** P < 0.001, **** P < 0.0001


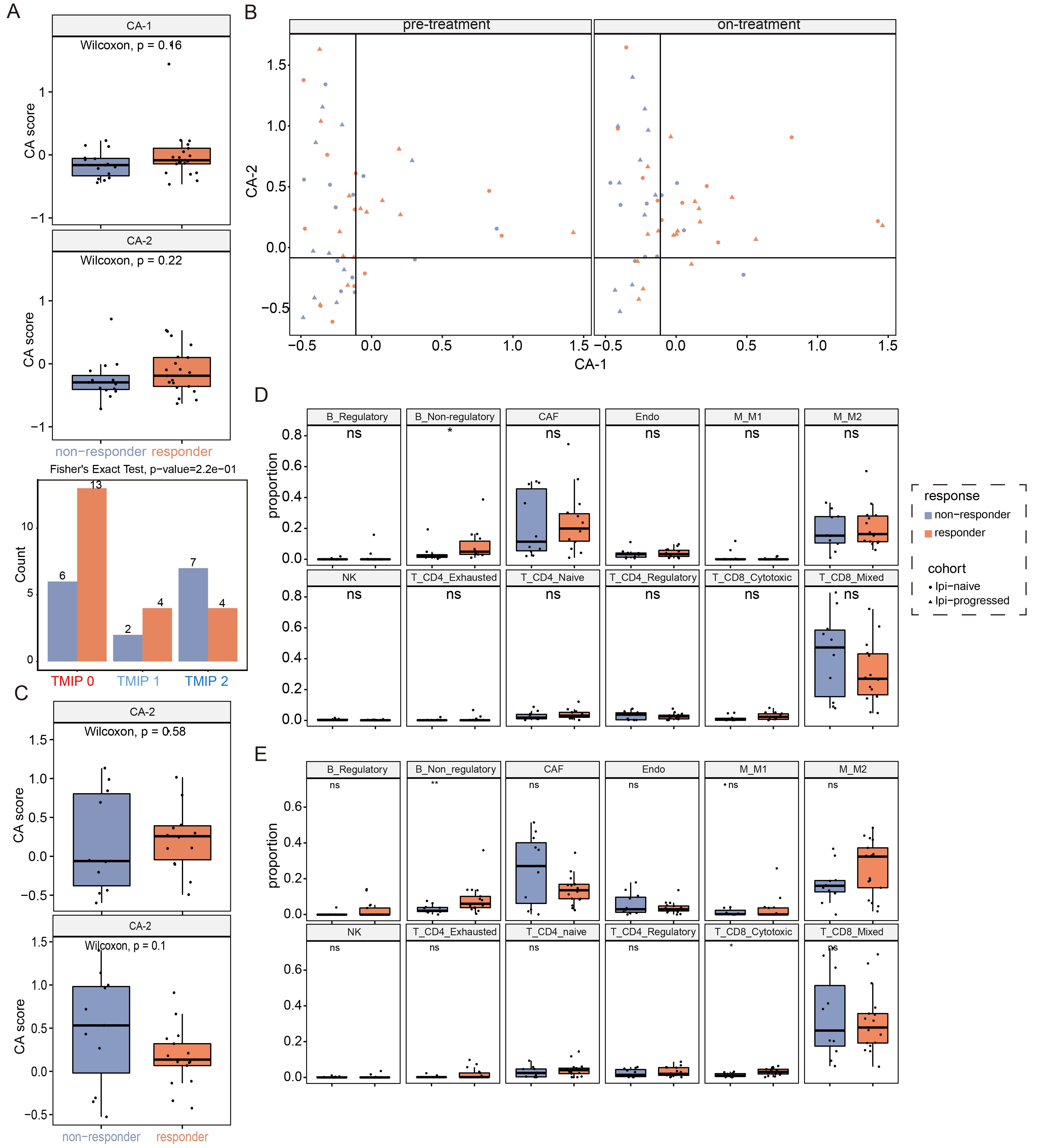
**Figure S7. Assessment on association between tumor microenvironment immune phenotypes (TMIP) and response to immune checkpoint blockade (ICB) in melanoma.** (A) Box plots showing differences of CA-1 (upper panel) and CA-2 (middle panel) scores between responders and non-responders in patients under immunotherapy in TCGA data. Bar charts showing numbers of responders and non-responders with different TMIPs in those patients (lower panel). (B) Projection of each patient of Riaz *et al.* dataset onto the first and second component of the correspondence analysis. Left panel showed pre-treatment samples and right panel denoted on-treatment patients. Non-responders were colored blue, and responders were colored orange. Points denoted Ipi-naive patients, and triangles denoted Ipi-progressed patients. (C) Box plots showing differences of CA-2 scores between responders and non-responders in anti-PD1 pre-treatment patients (upper panel) and on-treatment patients (lower panel) who progressed after a first-line anti-CTLA4 treatment (Ipi-progressed) in Riaz *et al.* data. (D-E) Comparison of each cell state proportion between responders and non-responders in Ipi-progressed patients based on pre-treatment (D) and on-treatment (E) transcriptomic profiles. ns: not significant; *: p < 0.05.

## 1.2 Supplementary Tables

**Table S1. Gene lists used for functional analyses.**

| **The relevant literatures surveyed to determine these key genes.** |
| --- |
| Single-cell RNA-seq enables comprehensive tumour and immune cell profiling in primary breast cancer. 2017 Nature communications |
| Single-Cell Map of Diverse Immune Phenotypes in the Breast Tumor Microenvironment. 2018 Cell |
| Immune Escape in Breast Cancer During In Situ to Invasive Carcinoma Transition. 2017 Cancer Discovery |
| Single-cell profiling of human gliomas reveals macrophage ontogeny as a basis for regional differences in macrophage activation in the tumor microenvironment. 2017 Genome Biology |
| Analysis of immune signatures in longitudinal tumor samples yields insight into biomarkers of response and mechanisms of resistance to immune checkpoint blockade. 2016 Cancer Discovery |
| Single-Cell Transcriptomic Analysis of Primary and Metastatic Tumor Ecosystems in Head and Neck Cancer. 2017 Cell |
| The Vigorous Immune Microenvironment of Microsatellite Instable Colon Cancer Is Balanced by Multiple Counter-Inhibitory Checkpoints. 2015 Cancer Discovery |
| Multiparametric profiling of non–small-cell lung cancers reveals distinct immunophenotypes. 2016 JCI Insight |
| Dissecting the multicellular ecosystem of metastatic melanoma by single-cell RNA-seq. 2016 Science |
| An Immune Atlas of Clear Cell Renal Cell Carcinoma. 2017 Cell |
| Landscape of Infiltrating T Cells in Liver Cancer Revealed by Single-Cell Sequencing. 2017 Cell |
| Global characterization of T cells in non-small-cell lung cancer by single-cell sequencing. 2018 Nature medicine |
| Single-cell profiling of breast cancer T cells reveals a tissue-resident memory subset associated with improved prognosis. 2018 Nature medicine |
| B cell regulation of the anti-tumor response and role in carcinogenesis. 2016 J Immunother Cancer |
| Factor XIIIA-expressing inflammatory monocytes promote lung squamous cancer through fibrin cross-linking. 2018 Nature communications |
| Robust enumeration of cell subsets from tissue expression profiles. 2015 Nature methods |

**Table S3. Demographics and characteristics of patients with melanoma.**

| Characteristic |  | No. of patients  (N=457) | No. of patients (Metastasis, N=362) |
| --- | --- | --- | --- |
| Age (years) | <50 | 134 | 120 |
|  | $\geq$ 50 | 315 | 234 |
|  | #N/A | 8 | 8 |
| Gender | FEMALE | 177 | 137 |
|  | MALE | 280 | 225 |
| Race | ASIAN | 11 | 5 |
|  | BLACK OR AFRICAN AMERICAN | 1 | 1 |
|  | WHITE | 435 | 348 |
|  | [Not Evaluated] | 7 | 6 |
|  | [Unknown] | 3 | 2 |
| Tumor Stage | Stage 0 | 7 | 7 |
|  | Stage I | 28 | 28 |
|  | Stage IA-B | 46 | 45 |
|  | Stage II | 30 | 26 |
|  | Stage IIA-C | 107 | 48 |
|  | Stage III | 41 | 39 |
|  | Stage IIIA-C | 125 | 103 |
|  | Stage IV | 22 | 20 |
|  | I/II NOS | 14 | 13 |
|  | [Not Available] | 37 | 33 |
| Tumor Status | TUMOR FREE | 208 | 138 |
|  | WITH TUMOR | 240 | 215 |
|  | #N/A | 9 | 9 |
| Tumor type | Primary | 95 | 0 |
|  | Metastasis | 362 | 362 |
| TCGA subtype | SKCM.- |  | 6 |
|  | SKCM.BRAF_Hotspot_Mutants |  | 114 |
|  | SKCM.NF1_Any_Mutants |  | 23 |
|  | SKCM.RAS_Hotspot_Mutants |  | 81 |
|  | SKCM.Triple_WT |  | 36 |

**Table S4. Uni- and multivariate analysis for progress-free survival (316 sample).**

| Variable |  | Univariate | | | Multivariate | | |
| --- | --- | --- | --- | --- | --- | --- | --- |
|  |  | HR | 95%CI | P value | HR | 95%CI | P value |
| Age | | 1.02 | 1.01-1.03 | < 0.0001* | 1.02 | 1.01-1.03 | 1e-04* |
| Gender | | | | | | | |
| MALE VS FEMALE | | 0.92 | 0.70-1.19 | 0.5087 | 0.91 | 0.70-1.19 | 0.4992 |
| Stage | | | | | | | |
| Stage I VS I/II NOS | | 2.04 | 0.87-4.75 | 0.1005 | 1.75 | 0.74-4.14 | 0.201 |
| Stage II VS I/II NOS | | 2.89 | 1.23-6.77 | 0.0149* | 2.11 | 0.88-5.05 | 0.0928 |
| Stage III VS I/II NOS | | 3.91 | 1.69-9.08 | 0.0015* | 3.52 | 1.50-8.26 | 0.0039* |
| Stage IV VS I/II NOS | | 5.44 | 2.03-14.57 | 7e-04* | 4.44 | 1.64-12.02 | 0.0033* |
| TMIP | | | | | | | |
| 1 VS 0 | | 1.46 | 1.07-1.98 | 0.016* | 1.32 | 0.96-1.82 | 0.0932 |
| 2 VS 0 | | 1.54 | 1.13-2.09 | 0.0061* | 1.70 | 1.24-2.34 | 0.001* |

CI, confidence interval; HR, hazard ratio.
*P < 0.05.

**Table S5. Uni- and multivariate analysis for overall survival (316 sample).**

| Variable |  | Univariate | | | Multivariate | | |
| --- | --- | --- | --- | --- | --- | --- | --- |
|  |  | HR | 95%CI | P value | HR | 95%CI | P value |
| Age | | 1.02 | 1.01-1.04 | <0.0001* | 1.02 | 1.01-1.03 | 4e-04* |
| Gender | | | | | | | |
| MALE VS FEMALE | | 1.06 | 0.77-1.47 | 0.7207 | 1.04 | 0.74-1.45 | 0.8258 |
| Stage | | | | | | | |
| Stage I VS I/II NOS | | 1.96 | 0.69-5.57 | 0.2058 | 1.65 | 0.57-4.77 | 0.3588 |
| Stage II VS I/II NOS | | 2.77 | 0.99-7.76 | 0.0534 | 1.98 | 0.69-5.70 | 0.2047 |
| Stage III VS I/II NOS | | 3.61 | 1.29-10.10 | 0.0143* | 3.09 | 1.08-8.81 | 0.0349* |
| Stage IV VS I/II NOS | | 4.81 | 1.38-16.79 | 0.0138* | 3.64 | 1.02-12.96 | 0.0457* |
| TMIP | | | | | | | |
| 1 VS 0 | | 1.80 | 1.24-2.62 | 0.002* | 1.58 | 1.07-2.34 | 0.0207* |
| 2 VS 0 | | 1.71 | 1.18-2.48 | 0.0044* | 1.74 | 1.19-2.56 | 0.0045* |

CI, confidence interval; HR, hazard ratio.
*P < 0.05.
